# Preparation of Monotrimethoxylsilylethyl-Terminated Polysiloxane Fluids and Their Application in Thermal Interface Materials

**DOI:** 10.3390/polym15163334

**Published:** 2023-08-08

**Authors:** Yang Liu, Xu Long, Yang Wang, Chuan Wu, Zhirong Qu, Zhiwei Pei, Chunlong Shi, Ting Wang, Hong Dong

**Affiliations:** 1 College of Material, Chemistry and Chemical Engineering, Key Laboratory of Organosilicon Chemistry and Material Technology, Ministry of Education, Hangzhou Normal University, Hangzhou 311121, Chinachuanwu@hznu.edu.cn (C.W.);; 2 Zhejiang Equation New Materials Co., Ltd., Quzhou 324000, China

**Keywords:** polymer matrix composites (PMCs), physical properties, surface treatments, thermal interface materials

## Abstract

In this study, *α*-Trimethylsilylmethyl-*ω*-dimethylsilyl-terminated polydimethylsiloxane, polydiethylsiloxane and poly[2,2,2-trifluoropropyl(methyl)siloxane] are synthesized using an anion catalyzed nonequilibrium polymerization reaction with trimethylsilylmethyl lithium as the initiator; hexamethylcyclotrisiloxane, hexaethylcyclotrisiloxane or 1,3,5-trimethyl-1,3,5-trifluoropropylcyclotrisiloxane as the monomer; and dimethylchlorosilane as an end-capping agent. Three kinds of *α*-trimethylsilylmethyl-*ω*-trimethoxylsilylethyl-terminated polysiloxanes are further prepared by hydrosilylation reaction of *α*-trimethylsilylmethyl-*ω*-dimethylsilyl-terminated polysiloxanes with vinyltrimethoxysilane using Karstedt’s catalyst. These *α*-trimethylsilylmethyl-*ω*-trimethoxylsilylethyl-terminated polysiloxanes are functionalized as in situ surface treatment agents for AlN particles. The effects of the structure of these polysiloxanes on the dispersion of AlN in the polysiloxane matrix and on the heat transfer performance of silicone pastes and silicone rubbers are investigated. A possible mechanism of surface treatment of AlN fillers by these novel silicone fluids is also discussed.

## 1. Introduction

In recent years, with the continuous development of microelectronic products toward high power and miniaturization, the heat dissipation performance of electronic components has become a key factor restricting the stability and reliability of electronic products. Although high-power electronic components are usually equipped with metal finned heat sinks for heat dissipation through heat conduction, there are still many extremely fine and uneven gaps existing between the contact surfaces of electronic components and finned heat sinks. Air with low thermal conductivity fills these gaps, making the actual effective contact area much smaller than its apparent value and resulting in a decrease in heat dissipation performance. Therefore, some soft and deformable materials with high thermal conductivity, namely thermal interface materials (TIMs), have been developed and placed in these gaps to enhance the heat transfer effect. TIMs can effectively eliminate air in gaps and reduce contact thermal resistance, timeously remove the heat generated during device operation and ensure the normal operation of the device [1,2,3]. Therefore, the thermal conductivity of TIMs plays a crucial role in the heat dissipation of electronic components. According to their product forms, TIMs can be divided into thermally conductive paste, thermally conductive pad, thermally conductive adhesive, thermally conductive gel, phase change material, etc. [4,5,6]. Although the characteristics, types and applicable locations of various TIMs are different, their main purpose is to reduce the effective thermal resistance of the heat dissipation path. Therefore, TIMs are crucial for filling these micropores to reduce the adverse effects of interfacial thermal resistance on heat transfer [7,8].

Polymers have the advantages of high electrical insulation, light weight, low cost and ease of processing, and have been widely used as electronic packaging materials for electronic devices [9]. However, the thermal conductivity of pure polymers is usually lower than 0.5 W/(m∙K), which greatly limits their application fields. Therefore, how to improve the thermal conductivity of polymers has become a focus for researchers and industry. Although various attempts have been made to design polymer chains to increase thermal conductivity [10,11], higher cost and complex processes are the two main obstacles hampering its practical application. In addition to designing the structure and morphology of the polymer itself, another effective method to improve the thermal conductivity of the polymer is to impregnate the polymer with thermally conductive fillers. Typically, thermally conductive fillers include metals [12], ceramics [13], carbon-based materials [14] and their mixtures [15]. Although thermal conductivity can be improved by adding metal or carbon-based material, the electrical conductivity of these TIMs has inevitably been increased simultaneously, which results in these TIMs not being able to be used in circumstances where high insulation performance is required. To obtain TIMs with good insulation performance, high thermal conductivity and good usability, surface treatment of the thermally conductive fillers is necessary. In this article, several monotrimethoxylsilylethyl-terminated polysiloxane fluids have been synthesized and characterized using NMR, GPC and TGA. The effect of these functional polysiloxane fluids on the surface treatment of AlN fillers and the heat transfer properties of the prepared silicone pastes is reported here.

## 2. Materials and Methods

### 2.1. Preparation of the Materials

Trimethylsilylmethyl lithium (TMSiMLi) was purchased from Meryer Biochemical Technology Co., Ltd. (Shanghai, China). Toluene and tetrahydrofuran (THF) with water content lower than 250 ppm were purchased from Sinopharm Chemical Reagent Co., Ltd. (Shanghai, China). Hexamethylcyclotrisiloxane (D_3_) with water content lower than 200 ppm was purchased from Hunan Huateng Pharmaceutical Co., Ltd. (Changsha, China). Hexaethylcyclotrisiloxane (D_3_^Et^) and 1,3,5-trimethyl-1,3,5-trifluoropropylcyclotrisiloxane (D_3_^F^) with water content lower than 200 ppm were purchased from Weihai Xinyuan New Materials Co., Ltd. (Weihai, China). Vinyl trimethoxysilane was purchased from Zhejiang Kaihua Synthetic Materials Co., Ltd. (Kaihua, China). Dimethylchlorosilane (DMCS) and *α*, *ω*-bistrimethylsilyl-terminated poly(dimethylmethylhydrogen)siloxane copolymer (a hydrogen-containing silicone fluid) with 0.1 wt% hydrogen content and *α*, *ω*-bisdimethylvinylsilyl-terminated polydimethylsiloxane (a terminal vinyl-containing silicone fluid) with a viscosity of 50 mPa.s at 25 °C were purchased from Jiaxing United Chemical Co., Ltd. (Jiaxing, China). AlN was purchased from Shanghai Bestry Performance Materials Co., Ltd. (Shanghai, China). Acetylene cyclohexanol (inhibitor) was purchased from ScienMax Inc. (Plano, TX, USA). Karstedt’s catalyst with 20.0 wt% platinum content was purchased from Heraeus Precious Metals Technology (China) Co., Ltd. (Shanghai, China) and diluted with toluene to 1% platinum content. All reagents were used as received.

#### 2.1.1. α-Trimethylsilylmethyl-ω-Trimethoxylsilylethyl-Terminated Polydimethylsiloxane (TMSM-PDMS-TMOS)

##### TMSM-PDMS-DMS

**Scheme 1:**
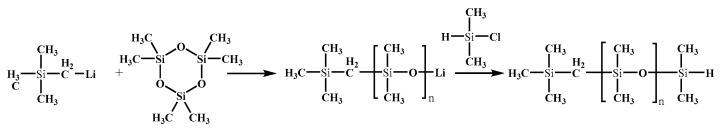
Synthesis of TMSM-PDMS-DMS.

Using D_3_ as the monomer, TMSiMLi as the anionic ring-opening polymerization initiator and DMCS as the end-capping agent, the preparation of TMSM-PDMS-DMS with a designed value of polymerization degree *n* = 10 is illustratively shown in Scheme 1. In a 1000 mL three-necked flask purged with drying nitrogen gas, 200 mL THF solution was added under stirring and cooled to 0 °C in an ice-water bath. Next, 220 mL hexane solution of TMSiMLi with a concentration of 0.56 mol/L was injected at a rate of 4 mL/min into the flask. Then, the mixture was maintained at 0 °C for about 30 min. Subsequently, the THF solution of D_3_ (100.01 g, 0.45 mol D_3_ dissolved in 160 mL THF) was added dropwise at a constant rate for 20 min, and then maintained at 0 °C for 30 min. The temperature of the mixture was then raised to 40 °C and maintained for 6 h. When thin-layer chromatography (TLC) showed that all the raw materials had disappeared, 15.09 g (0.149 mol) of DMCS was injected into the reactor for about 60 min to terminate the reaction. When most of the low-boiling solvents such as THF were removed from the mixed solution containing insoluble substances by flash distillation at atmospheric pressure, the mixture was cooled to room temperature, and 105.22 g of filtrate was collected by filtration. The filtrate was then distilled under reduced pressure (absolute pressure 0.2 kPa) at 180 °C for 3 h to remove low-molecular-weight substances, which resulted in 101.98 g of the product after cooling to room temperature. When TMSiMLi was used as the key component, the total yield of TMSM-PDMS-DMS was estimated to be 83.0%.

##### TMSM-PDMS-TMOS

**Scheme 2:**
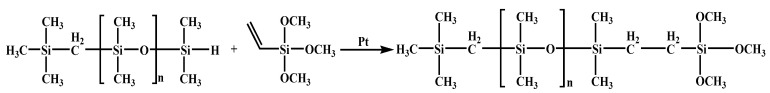
Synthesis of TMSM-PDMS-TMOS by hydrosilylation reaction.

Taking a polymer with a designed value of *n* = 10 as an example, the preparation process of TMSM-PDMS-TMOS is illustratively shown in Scheme 2. A solution consisting of 80 mL toluene with a water content of 220 ppm, 23.98 g (0.162 mol) vinyltrimethoxysilane and 0.2426 g of 1 wt% Karstedt’s catalyst with an effective Pt concentration of 20 ppm was added to a 500 mL three-necked flask purged with dry nitrogen, stirred and heated to 70 °C. The Karstedt’s catalyst was activated at this temperature for 30 min. Next, 98.19 g (0.108 mol) TMSM-PDMS-DMS with molar mass *M*_NMR_ = 908 g/mol and *n* = 10.3 detected using ^1^H NMR spectroscopy was added to the reaction mixture through a constant-pressure drop funnel for 1 h. Then, the mixture was reacted at 70 °C for 3 h. The reaction mixture was cooled to room temperature and the catalyst was deactivated. After removing the low-boiling substances by vacuum distillation, 110.90 g of the product with a yield of 97.0% relative to TMSM-PDMS-DMS was obtained.

#### 2.1.2. α-Trimethylsilylmethyl-ω-Trimethoxylsilylethyl-Terminated Poly[2,2,2-Trifluoropropyl(methyl)siloxane] (TMSM-PTFPMS-TMOS)

##### TMSM-PTFPMS-DMS

**Scheme 3:**
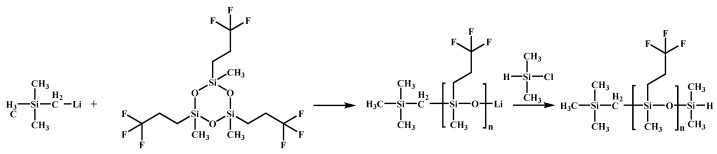
Synthesis of TMSM-PTFPMS-DMS.

Taking a polymer with a designed value of *n* = 10 as an example, the synthesis of TMSM-PTFPMS-DMS is illustratively shown in Scheme 3. In a 250 mL three-necked flask purged with drying nitrogen gas, 50 mL THF solution was added under stirring and cooled to 0 °C in an ice-water bath. Next, 57 mL of a hexane solution of TMSiMLi with a concentration of 0.56 mol/L was injected at a rate of 4 mL/min into the flask. Then, the mixture was maintained at 0 °C for about 30 min. Subsequently, 50.01 g (0.107 mol) of D_3_^F^ was dropped at a constant rate for 30 min, and then maintained at 0 °C for another 30 min after dropping. The temperature of the mixture was then raised to 30 °C and maintained for 20 h. When TLC showed that all the raw materials had disappeared, 3.72 g (0.035 mol) of DMCS was injected into the reactor and the condensation reaction lasted for about 60 min. When most of the low-boiling solvents such as THF were removed from the mixed solution containing insoluble substances by flash distillation at atmospheric pressure, the mixture was cooled to room temperature and 44.23 g of filtrate was collected by filtration. The filtrate was then distilled under reduced pressure (absolute pressure 0.2 kPa) at 180 °C for 3 h to remove low-molecular-weight substances, which resulted in 41.19 g of the product after cooling to room temperature. When TMSiMLi was used as the key component, the total yield of TMSM-PTFPMS-DMS was estimated to be 75.0%.

##### TMSM-PTFPMS-TMOS

**Scheme 4:**
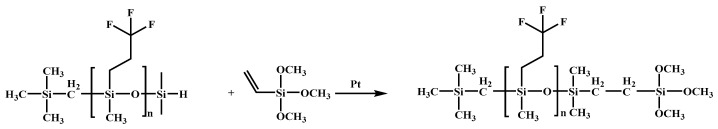
Synthesis of TMSM-PTFPMS-TMOS by hydrosilylation reaction.

Taking a polymer with a designed value of *n* = 10 as an example, the preparation process of TMSM-PTFPMS-TMOS is illustratively shown in Scheme 4. A solution consisting of 15 mL toluene with a water content of 220 ppm, 4.44 g (0.0297 mol) of vinyltrimethoxysilane and 1.0222 g of 1 wt% Karstedt’s catalyst with effective Pt concentration of 30 ppm were added to a 250 mL three-necked flask purged with drying nitrogen, stirred and heated to 70 °C. The Karstedt’s catalyst was activated at this temperature for 30 min. Next, 27.60 g (0.0198 mol) of TMSM-PTFPMS-DMS with molar mass *M*_NMR_ = 1628 g/mol and *n* = 9.5 detected using ^1^H NMR spectra was added to the reaction mixture through a constant-pressure drop funnel for 1 h. Then, the mixture was reacted at 70 °C for 3 h. The reaction mixture was cooled to room temperature and the catalyst was deactivated. After removing the low-boiling substances by vacuum distillation, 25.01 g of the product with a yield of 83.0% relative to TMSM-PTFPMS-DMS was obtained.

#### 2.1.3. α-Trimethylsilylmethyl-ω-Trimethoxylsilylethyl-Terminated Polydiethylsiloxane (TMSM-PDES-TMOS)

##### TMSM-PDES-DMS

**Scheme 5:**
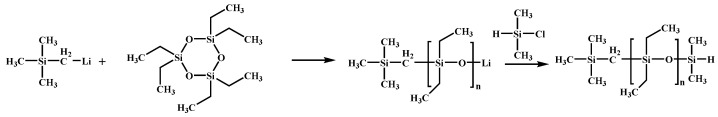
Synthesis of TMSM-PDES-DMS.

Taking a polymer with a designed value of *n* = 10 as an example, the synthesis of TMSM-PDES-DMS is illustratively shown in Scheme 5. In a 250 mL three-necked flask purged with drying nitrogen gas, 50 mL THF solution was added under stirring and cooled to 0 °C in an ice-water bath. Next, 88 mL of a hexane solution of TMSiMLi with a concentration of 0.56 mol/L was injected at a rate of 4 mL/min into the flask. Then, the mixture was maintained at 0 °C for about 30 min. Subsequently, 50.02 g (0.163 mol) of D_3_^Et^ was dropped at a constant rate for about 60 min and then maintained at 0 °C for another 30 min after dropping. The temperature of the mixture was then raised to 40 °C and maintained for 24 h. When TLC showed that all the raw materials had disappeared, 5.12 g (0.054 mol) of DMCS was injected into the reactor and the condensation reaction lasted for about 60 min. When most of the low-boiling solvents such as THF were removed from the mixed solution containing insoluble substances by flash distillation at atmospheric pressure, the mixture was cooled to room temperature and 30.41 g of filtrate was collected by filtration. The filtrate was then distilled under reduced pressure (absolute pressure 0.2 kPa) at 180 °C for 3 h to remove low-molecular-weight substances, which resulted in 16.50 g of the product after cooling to room temperature. When TMSiMLi was used as the key component, the total yield of TMSM-PDES-DMS was estimated to be 28.0%.

##### TMSM-PDES-TMOS

**Scheme 6:**
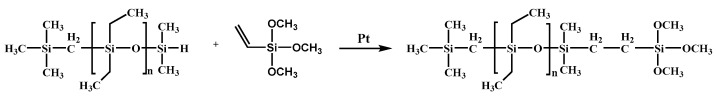
Synthesis of TMSM-PDES-TMOS by hydrosilylation reaction.

Taking a polymer with a designed value of *n* = 10 as an example, the preparation process of TMSM-PDES-TMOS is illustratively shown in Scheme 6. A solution consisting of 10 mL toluene with a water content of 220 ppm, 2.10 g (0.0413 mol) of vinyltrimethoxysilane and 0.4233 g of 1 wt% Karstedt’s catalyst with effective Pt concentration of 30 ppm were added to a 100 mL three-necked flask purged with dry nitrogen, stirred and heated to 70 °C. The Karstedt’s catalyst was activated at this temperature for 30 min. Next, 12.02 g (0.010 mol) of TMSM-PDES-DMS with molar mass *M*_NMR_ = 1268 g/mol and *n* = 11 detected using ^1^H NMR spectra was added to the reaction mixture through a constant-pressure drop funnel for 1 h. Then, the mixture was reacted at 70 °C for 3 h. The reaction mixture was cooled to room temperature and the catalyst was deactivated. After removing the low-boiling substances by vacuum distillation, 11.89 g of the product with a yield of 89.0% relative to TMSM-PDES-DMS was obtained.

### 2.2. Charactrization

For liquid samples, an AvanceAV 400 NMR spectrometer (Bruker, Fällanden, Switzerland) was used in addition to a solid–liquid dual-purpose AvanceAV III 500 NMR spectrometer (Bruker, Fällanden, Switzerland). The ^1^H NMR was obtained by dissolving the liquid sample in deuterated chloroform (CDCl_3_) solvent without internal standards and measured in an AvanceAV 400 NMR spectrometer. The ^13^C NMR, ^29^Si NMR and ^19^F NMR spectra were measured and recorded in the AvanceAV III 500 spectrometer by dissolving the liquid sample in CDCl_3_ solvent without internal standards. Fourier-transform infrared (FT-IR) spectroscopy was recorded in a Bruker Alpha-T (Ettlingen, Germany) with a resolution of 4 cm^−1^, 64 scans and a range of 4000–400 cm^−1^. The molecular weights of polymers were determined using a PL-GPC50 gel-permeation chromatograph (GPC) from Agilent (CA, USA), with polydimethylsiloxane (American Polymer Standards Company, OH, USA) as the standard sample, toluene as the mobile phase at a flow rate of 1.0 mL/min and column temperature of 35 °C. A thermogravimetric analyzer (TGA), Discovery TGA (TA Instruments, DE, USA), was used under a nitrogen atmosphere with a gas flow rate of 30 mL/min, heating rate of 20 K/min and a temperature range between 40 and 800 °C. A Netzsch DSC 214 differential scanning calorimeter (DSC; NETZSCH, Wittelsbacherstrasse, Germany) was used under a nitrogen atmosphere (40 mL/min) and the temperature of the samples was first lowered from room temperature to −140 °C at a rate of 10 K/min, and then heated at a rate of 10 K/min to 25 °C. The thermal conductivity and thermal resistance of the composites were tested according to the ASTM D5470 method using a DRL-III thermal conductivity tester produced by Xiangtan Xiangyi Instrument Co., Ltd. (Xiangtan, China). The heating plate was set at 50 °C, and the sample thickness was 1–2 mm. The surface of the composites was observed using a SIGMA 500 Zeiss scanning electron microscope (Jena, Germany) at testing voltage of 3 kV in a vacuum of 5.4 × 10^−8^ Pa.

AlN fillers were mixed with silicone fluids and other liquid samples in a noninterventional material homogenizer (Planetary Mixer, ZYMC–180HV; Shenzhen ZYE Technology Co., Ltd., Shenzhen, China).

In the Supporting Information, the structural information of these three kinds of monotrimethoxylsilylethyl-terminated polysiloxane fluids and each of their intermediates is verified by NMR spectra (Figures S1–S3 for TMSM-PDMS-DMS, Figures S12–S14 for TMSM-PTFPMS-DMS, Figures S25–S27 for TMSM-PDES-DMS, Figures S7–S9 for TMSM-PDMS-TMOS, Figures S19–S22 for TMSM-PTFPMS-TMOS, and Figures S32–S34 for TMSM-PDES-TMOS) and FT-IR spectra (Figure S4 for TMSM-PDMS-TMOS, Figure S15 for TMSM-PTFPMS-DMS, Figure S28 for TMSM-PDES-DMS, Figure S10 for TMSM-PDMS-TMOS, Figure S23 for TMSM-PTFPMS-TMOS, and Figure S35 for TMSM-PDES-TMOS). These results indicated that the investigated monotrimethoxylsilylethyl-terminated polysiloxane fluids and their intermediates were successfully prepared in this study. The thermal performance of these three kinds of polymers were also tested by TGA (Figure S6 for TMSM-PDMS-DMS, Figure S16 for TMSM-PTFPMS-DMS, Figure S29 for TMSM-PDES-DMS, Figure S11 for TMSM-PDMS-TMOS, Figure S24 for TMSM-PTFPMS-TMOS, and Figure S36 for TMSM-PDES-TMOS) and DSC (Figure S17 for TMSM-PTFPMS-DMS and Figure S30 for TMSM-PDES-DMS) and are presented in the Supporting Information. GPC curves of TMSM-PDMS-DMS, TMSM-PTFPMS-DMS and TMSM-PDES-DMS were presented in Figures S5, S18 and S31, respectively. The molecular characteristics of all the samples prepared in this study are summarized in Table 1. Since terminal methoxy groups were highly active, attempts to obtain accurate GPC results failed. Therefore, only molecular weights calculated from ^1^H NMR (MNMR) are presented for TMSM-PDMS-TMOS, TMSM-PTFPMS-TMOS and TMSM-PDES-TMOS in Table 1.

Although the molecular weight of a polymer could be determined either by GPC or ^1^HNMR spectra, the results obtained from these two methods were hard to compare directly because their measurement principles were completely different. The accuracy of the GPC results depended on the mobile phase and the molecular weight data of the reference material provided by the manufacturer, and the molecular weight of the polymer calculated by ^1^H NMR spectra was highly dependent on the integrated peak area of each kind of protons in Si-OCH_3_ (or Si-H) and Si-CH_3_ group in the polymer with known structure.

### 2.3. Preparation of Thermally Conductive Composites

#### 2.3.1. Thermally Conductive Paste

Terminal vinyl-containing silicone fluid and one of the three kinds of functional polymers containing terminal methoxy groups prepared in this manuscript, namely, TMSM-PDMS-TMOS, TMSM-PTFPMS-TMOS, or TMSM-PDES-TMOS, were mixed at a mass ratio of 2:1 or 9:1 (when TMSM-PDES-TMOS was used) in a 100 mL mixing cup at room temperature. After stirring evenly, the mixing cup was put into a noninterventional material homogenizer for stirring and defoaming treatment (1800 rpm, −99.5 kPa) for 3 min.

AlN fillers with three average particle sizes (50, 20 and 1 μm) were weighed in a mass ratio of 9:5:3 and then mixed with the above polysiloxane mixtures in the mixing cup and then put in a noninterventional material homogenizer. After stirring and defoaming at 1800 rpm and −99.5 kPa for 5 min, thermally conductive silicone paste could be prepared. By changing the ratio of AlN fillers to polysiloxane mixtures, thermally conductive silicone paste samples with different filler loads could be prepared.

#### 2.3.2. Thermal Silicone Rubber

In a 100 mL mixing cup, a certain mass of terminal vinyl-containing silicone fluid with a viscosity of 50 mPa.s at 25 °C was mixed with one of the three kinds of silicone fluids, namely, TMSM-PDMS-TMOS, TMSM-PTFPMS-TMOS, or TMSM-PDES-TMOS, in a mass ratio of 2:1 and mixed homogeneously in a planetary mixer. After that, Karstedt’s catalyst with 10 ppm platinum content and the inhibitor ethynyl cyclohexanol with 10 ppm concentration were added to the mixing cup, stirred and defoamed at 1800 rpm and −99.5 kPa for 3 min in a planetary mixer to ensure that all materials were mixed evenly.

A certain amount of AlN fillers consisting of three average particle sizes of 50 μm, 20 μm and 1 μm were added in a mass ratio of 9:5:3 to the previously prepared homogeneous liquid mixture, stirred and defoamed at 1800 rpm and −99.5 kPa in a planetary mixer for 5 min to ensure all AlN fillers was evenly dispersed in the liquid mixture.

A certain mass of hydrogen-containing silicone fluid with 0.1 wt% mass fraction of hydrogen content was added in a ratio of Si–H/Si–Vi = 1.4:1 (molar ratio) to the mixing cup and functioned as a cross-linking agent for the hydrosilylation reaction. Then, the mixing cup was placed in a planetary mixer, stirred and defoamed at 1800 rpm and −99.5 kPa for 5 min. The uniformly mixed material was then put into an oven at 120 °C for 1 h to obtain a thermally conductive silicone rubber composite.

## 3. Results and Discussion

### 3.1. Effect of Reaction Time on the Yield of TMSM-PDMS-DMS

Keeping the amount of all the chemicals unchanged, the yield of TMSM-PDMS-DMS varied with the reaction time, and the results are shown in Table 2.

During the ring-opening polymerization of cyclotrisiloxane catalyzed by anion, to avoid the chain transfer reaction or equilibrium polymerization of the generated polymer, it was usually necessary to conduct the polymerization at a low temperature. When the reaction temperature was determined, the reaction time became the key factor affecting the yield of the polymer and the molecular weight of the polymer and its distribution. To investigate the effect of reaction time on the yield, molecular weight and molecular weight distribution of the obtained product, the designed polymeric degree was set to *n* = 10 and the amount of each material was fixed. The influence of the polymerization process of D_3_ initiated by TMSiMLi is shown in Table 2 as an example. It was found that the yield reached the highest value when the time was set to 6 h. If the reaction time was extended, the nonequilibrium polymerization reaction initiated by TMSiMLi would develop into the equilibrium polymerization stage, which would result in the rearrangement of the molecular weight of the polymer and broaden the molecular weight distribution [16]. If the reaction time was shortened, although the molecular weight distribution of the prepared polymer was narrow, large amounts of cyclotrisiloxanes failed to participate in the ring-opening polymerization reaction and resulted in low monomer conversion.

### 3.2. Effect of Solvent Types on the Yield of TMSM-PTFPMS-DMS

Compared with traditional polydimethylsiloxane (PDMS), silicon materials containing fluorine elements not only retain the typical heat- and cold-resistant properties, flexibility, radiation resistance and excellent electrical properties of PDMS but also can effectively improve the oil resistance and low dielectric constant characteristics of organosilicon materials through the introduction of fluorine elements [17]. Based on the successful preparation of TMSM-PDMS-DMS, the synthetic conditions for TMSM-PTFPMS-DMS were explored when monomer D_3_ was substituted by D_3_^F^.

It has been reported in the literature that the ring-opening polymerization reaction of D_3_^F^ was greatly affected by the solvent. Therefore, other solvents apart from THF were also investigated in the ring-opening polymerization reaction of D_3_^F^ under the same reaction conditions to reveal the effect of the solvent type and molecular structure on the yield of TMSM-PTFPMS-DMS. The results are shown in Table 3.

As can be seen from Table 3, the solvent had a significant impact on the product yield. Among the three solvents considered, the product yield was highest when using THF, so THF was used as the solvent in the ring-opening polymerization reaction of D_3_^F^.

### 3.3. Effect of Water Content and Monomer Structure on Yield of Polymer

For the anionic ring-opening polymerization of six-membered cyclic siloxane initiated by TMSiMLi, no matter which kind of cyclotrisiloxane is selected as the polymer monomer, the influence of moisture on the yield of the synthetic product is very significant because the moisture in the system easily reacted with the alkyl lithium and deactivated it. Therefore, the moisture in the system and raw materials must be strictly controlled during the experiment.

Compared with the methyl and trifluoropropyl groups, the introduction of ethyl groups into the side chains of polysiloxane will effectively improve the low-temperature performance and friction resistance of silicone materials. Although the synthesis of monodimethylbutylsiloxyl-terminated polydiethylsiloxane has been reported in the literature using *n*-butyllithium as an anionic nonequilibrium ring-opening polymerization initiator and D_3_^Et^ as a polymeric monomer [18], the nonequilibrium ring-opening polymerization of D_3_^Et^ initiated by TMSiMLi has rarely been reported. Therefore, apart from TMSM-PDMS-DMS and TMSM-PTFPMS-DMS, the feasibility of synthesizing TMSM-PDES-DMS was also explored using D_3_^Et^ as the polymer monomer and TMSiMLi as the initiator for the nonequilibrium ring-opening polymerization process. Compared with D_3_ and D_3_^F^, it was found that the yield of the nonequilibrium polymerization of D_3_^Et^ was very low. Probably because of the lower activity of D_3_^Et^, Tsuchihara et al. reported that D_3_^Et^ could not form a polymer in the hexane solution of *n*-butyllithium when no activator was used [19]. Only in an anhydrous environment and protected by argon gas could the ring-opening polymerization of D_3_^Et^ be conducted in the presence of activators such as crown ether [19], hexamethylphosphorictriamide, (*n*-BuO)_3_PO, THF, DMSO or NMP to form polymers [20,21].

### 3.4. Effect of Polysiloxane Structure on Thermal Conductivity of AlN-Filled Composites

#### 3.4.1. TMSM-PDMS-TMOS

**Figure 1:**
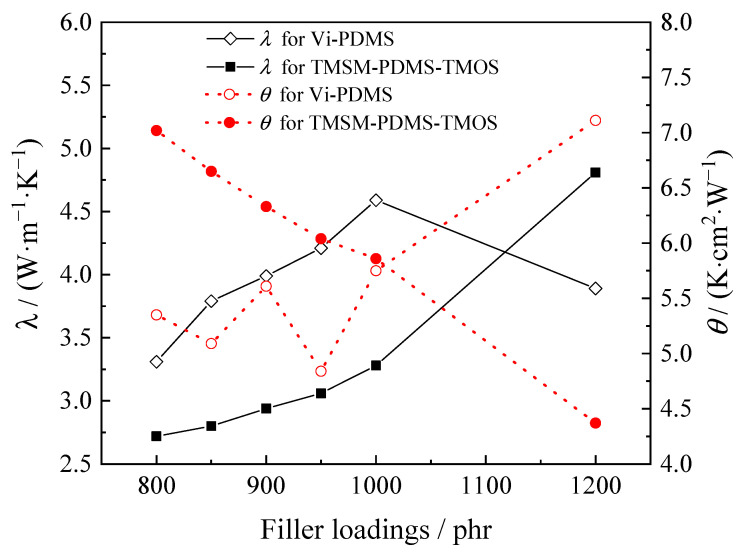
Effect of untreated or TMSM-PDMS-TMOS-treated AlN hybrid fillers on thermal conductivity and thermal resistance of composites (terminal vinyl-containing silicone fluid (Vi-PDMS) with a viscosity of 50 mPa.s at 25 °C used alone or mixed with TMSM-PDMS-TMOS with a viscosity of 10 mPa.s at 25 °C in a mass ratio of 2:1. AlN hybrid fillers consist of particles with average particle size of 50 μm, 20 μm and 1 μm in a mass ratio of 9:5:3).

The synthesized TMSM-PDMS-TMOS was used as the in situ surface treatment agent for AlN fillers with different particle sizes. The variation of thermal conductivity (*λ*) and thermal resistance (*θ*) of AlN-filled composites with the amount of AlN mixed filler for pure terminal vinyl-containing silicone fluid (Vi-PDMS) and that of the mixture of Vi-PDMS and TMSM-PDMS-TMOS is shown in Figure 1.

It can be seen from Figure 1 that when loadings of mixed AlN fillers were lower than 1000 parts (relative to 100 parts of pure Vi-PDMS or its mixture with TMSM-PDMS-TMOS), the thermal conductivities of the silicone pastes prepared either from pure Vi-PDMS or its mixture with TMSM-PDMS-TMOS gradually increase with the increase in filler loadings. Meanwhile, the thermal resistance of these two kinds of silicone pastes exhibited a downward trend with the increase in filler loadings. When the loadings exceeded 1000 parts, the thermal conductivity of silicone paste prepared from pure Vi-PDMS decreased sharply. For example, when dosage of the mixed AlN fillers increased from 1000 parts to 1200 parts, the thermal conductivity of this kind of silicone paste decreased from 4.59 W/(m∙K) to 3.89 W/(m∙K), while its thermal resistance increased from 5.75 K∙cm^2^/W to 7.11 K∙cm^2^/W. However, the thermal conductivity of silicone paste prepared from the mixture of Vi-PDMS and TMSM-PDMS-TMOS exhibited different trends. With the filler loadings increased, its thermal conductivity increased steadily and its thermal resistance decreased steadily. When the dosage of AlN fillers reached 1200 parts, the thermal conductivity of this kind of silicone paste reached 4.81 W/(m∙K), which was 0.92 W/(m∙K) higher than that of silicone paste prepared from pure Vi-PDMS, together with a thermal resistance reduced by 2.74 K∙cm^2^/W.

It can also be seen from Figure 1 that when the loadings of mixed AlN fillers were lower than 1000 parts, the thermal conductivity of the silicone paste prepared from Vi-PDMS was always higher than that of the silicone paste prepared from the mixture of Vi-PDMS and TMSM-PDMS-TMOS, and the thermal resistance of the former was somewhat smaller than that of the latter. This phenomenon might be related to the presence of hydrophilic hydroxyl groups on the surface of AlN, which made AlN poorly compatible with hydrophobic Vi-PDMS. When the loading of AlN fillers was low, although these AlN fillers with mixed particle sizes could not be uniformly dispersed in a relatively large amount of Vi-PDMS, these AlN fillers could still be packed together in Vi-PDMS matrix under the “bonding” influence of Vi-PDMS and formed a thermally conductive pathway. As the amount of filler increased, the number of thermal conduction channels formed increased, and the thermal conductivity of the silicone paste increased accordingly. However, the viscosity of the silicone paste increased sharply with the increase in the amount of filler because the hydroxyl groups on the surface of the filler were not compatible with hydrophobic Vi-PDMS matrix. For example, when using 100 parts of Vi-PDMS with a viscosity of 50 mPa.s as the matrix, the viscosities of silicone pastes filled with 800, 850 and 900 parts of mixed-size AlN particles were 11,980, 16,250 and 28,200 mPa.s, respectively. When the amount of filler reached 1200 parts, the filler could not be fully wetted by the Vi-PDMS at this time. Some air with lower thermal conductivity filled the inside of the silicone paste and resulted in a sharp decrease in the thermal conductivity of the silicone paste.

When the mixture of Vi-PDMS and TMSM-PDMS-TMOS was used instead of pure Vi-PDMS as the matrix of silicone paste, the terminal methoxy groups in TMSM-PDMS-TMOS reacted with the hydroxyl group on the surface of the AlN fillers through condensation reaction and reduced the content of hydroxyl groups on the surface of the AlN filler. Meanwhile, a layer of trimethylsilylmethyl-terminated PDMS could graft onto the surface of the AlN fillers and greatly enhanced the compatibility between AlN fillers and the polymer matrix. Therefore, compared with untreated AlN fillers in Vi-PDMS, these surface treated AlN particles could uniformly disperse in polysiloxane matrix under the same or even higher AlN filler loadings. For example, when one of three parts of Vi-PDMS was substituted by TMSM-PDMS-TMOS, the viscosities of silicone pastes filled with 800, 850 and 900 parts of mixed-size AlN particles were 7075, 11,920 and 22,237 mPa.s, respectively. Compared with pure Vi-PDMS as the matrix of silicon paste, the viscosity of each silicone paste decreased by 40.94%, 26.65% and 21.15%, respectively.

Because each AlN particle was covered by a layer of silicone material with a lower thermal conductivity than AlN itself, the silicone paste prepared in this method was not a good conductor of heat. Although the silicone paste prepared under such small AlN filler loadings had lower viscosity and was easier to coat, its thermal conductivity was inferior to that of silicone paste prepared by untreated AlN fillers and pure Vi-PDMS, because the random packing of untreated AlN fillers in Vi-PDMS matrix might be beneficial to heat transfer.

With the increase in AlN filler loadings, these surface treated AlN fillers could still be uniformly dispersed in polysiloxane matrix since they had good compatibility with silicone materials. Through the close packing among these surface treated AlN fillers, more heat conduction channels were formed inside the silicone paste. Therefore, the thermal conductivity of the silicone paste prepared with TMSM-PDMS-TMOS-treated AlN fillers tended to increase gradually. In contrast, the viscosity of the silicone paste prepared by untreated AlN fillers and pure Vi-PDMS increased sharply with the increase in AlN filler loadings. When the amount of AlN filler increased to 1200 parts, some fillers could not be uniformly dispersed in Vi-PDMS. The hard silicone paste was damaged and could not flow at all. At this time, the air with the lowest thermal conductivity filled inside the silicone paste and the established heat transfer channels were destroyed, which resulted in a sharp decrease in the thermal conductivity of the silicone paste.

Although the dosage of surface treated AlN filler could be further increased and the added AlN filler could still be evenly dispersed into the polysiloxane matrix, the resulting composite became relatively hard, and no fluidity could be maintained. Composites prepared with these large amounts of AlN fillers could not be functionalized as thermal pastes because they did not have the coating properties and extensibility required for thermal pastes.

#### 3.4.2. TMSM-PTFPMS-TMOS

**Figure 2:**
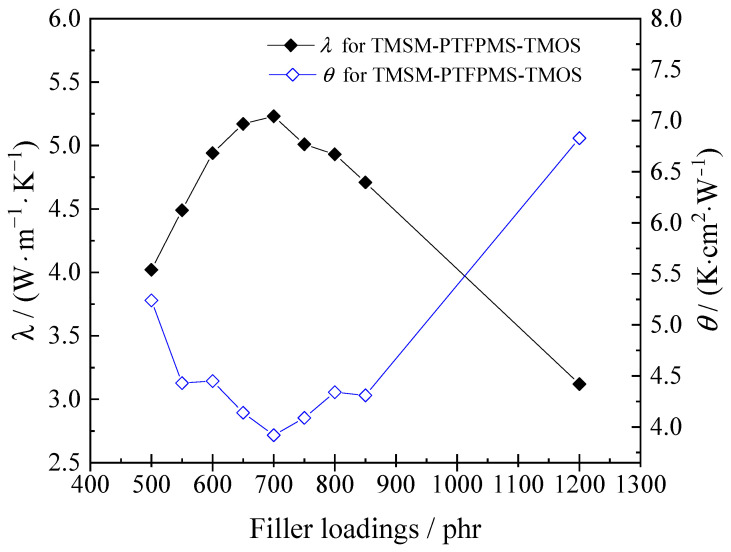
Effect of TMSM-PTFPMS-TMOS-treated AlN mixed fillers on the thermal conductivity and thermal resistance of the composites (terminal vinyl-containing silicone fluid with a viscosity of 50 mPa.s at 25 °C and TMSM-PTFPMS-TMOS with a viscosity of 10 mPa.s at 25 °C were mixed at a mass ratio of 2:1. AlN fillers with an average particle size of 50 μm, 20 μm and 1 μm were mixed at a mass ratio of 9:5:3).

When TMSM-PDMS-TMOS was substituted by TMSM-PTFPMS-TMOS with a viscosity of 10 mPa.s at 25 °C and functioned as the surface treatment agent for mixed AlN fillers, the composites were prepared according to the method described above. The effect of TMSM-PTFPMS-TMOS on AlN fillers, on the thermal conductivity and thermal resistance of AlN filler-filled composites is shown in Figure 2.

It can be seen from Figure 2 that the thermal conductivity of the composite increased with the dosage of AlN filler loadings changed from 500 parts to 700 parts, and the thermal resistance of this composite decreased with the dosage of AlN filler loadings. When the amount of AlN filler treated with TMSM-PTFPMS-TMOS reached 700 parts, the thermal conductivity of the composite reached 5.23 W/(m∙K) and its thermal resistance reached 3.92 K∙cm^2^/W. When the amount of the treated AlN filler exceeded 700 parts, the thermal conductivity of the silicone paste tended to decrease drastically and its thermal resistance increased quickly. The reason that the thermal conductivity of the composite materials sharply decreased and its thermal resistance drastically increased with the increase in filler loadings might be directly related to the low polarizability and strong electronegativity of fluorine atoms. On the other hand, fluorine-containing polysiloxanes had lower surface tension. The methoxy group on the terminal group of TMSM-PTFPMS-TMOS could undergo a condensation reaction with the hydroxyl group on the surface of the AlN filler, making the inert trimethylsilylmethyl-terminated poly[2,2,2-trifluoropropyl(methyl)siloxane] chains grafted onto the AlN surface. However, because of the significant steric hindrance of the polymer, we suspected that not all the hydroxyl groups on the AlN surface could be completely modified by TMSM-PTFPMS-TMOS. Under such circumstances, the trifluoropropyl functional groups that had been grafted on the AlN surface might strongly repel other AlN filler particles with residual hydroxyl groups, resulting in the inability to achieve close packing among surface-modified fillers or an even distribution of surface-modified filler particles in the PDMS matrix. Therefore, the thermal conductivity pathway was interrupted and thus the thermal conductivity of the composites decreased sharply. As the amount of AlN fillers increased, the number of AlN fillers with residual hydroxyl groups or untreated hydroxyl groups inside the composite material also increased. Under the strong repulsion of trifluoropropyl groups, these AlN fillers were difficult to disperse evenly and formed thermal conductivity pathways inside the composite material. Therefore, the thermal conductivities of the composites showed a sharp decreasing trend, while their thermal resistances exhibited a quick increasing trend with the increase in AlN fillers.

#### 3.4.3. TMSM-PDES-TMOS

Because of the high price and low yield of the ring-opening polymerization reaction of D_3_^Et^, to evaluate the effect of TMSM-PDES-TMOS on the treatment of AlN fillers, on the thermal conductivity and thermal resistance of composites filled with surface-modified AlN fillers, terminal vinyl-containing silicone fluid with a viscosity of 50 mPa.s at 25 °C was mixed with TMSM-PDES-TMOS with a viscosity of 10 mPa.s at 25 °C in a mass ratio of 9:1. When different amounts of AlN fillers with an average particle size of 50, 20 and 1 μm were mixed with the siloxane mixtures at a mass ratio of 9:5:3, silicone pastes with different dosages of modified AlN fillers were prepared.

**Figure 3:**
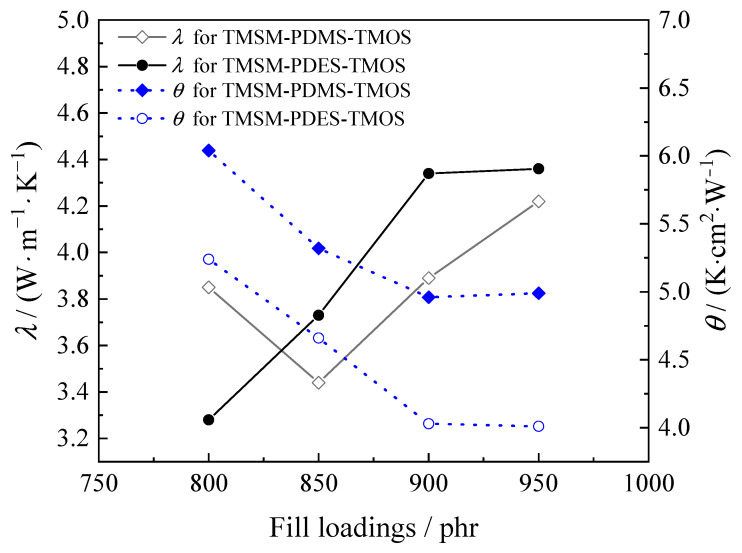
The effect of TMSM-PDMS-TMOS or TMSM-PDES-TMOS on AlN fillers and their filled silicone pastes (50 mPa.s terminal vinyl-containing silicone fluid mixed with TMSM-PDMS-TMOS or TMSM-PDES-TMOS in a mass ration of 9:1, AlN fillers with an average particle size of 50 μm, 20 μm and 1 μm were mixed at a mass ratio of 9:5:3).

To compare the influence of polysiloxane structure on the thermal conductivities and thermal resistances of composites, TMSM-PDES-TMOS was replaced with TMSM-PDMS-TMOS with the same viscosity while other parameters remained the same. Silicone pastes were prepared, and their thermal conductivities and thermal resistances are also presented in Figure 3 for comparison with those obtained with TMSM-PDES-TMOS.

As shown in Figure 3, when terminal vinyl-containing silicone fluid with a viscosity of 50 mPa.s at 25 °C was mixed with TMSM-PDMS-TMOS or TMSM-PDES-TMOS in a mass ratio of 9:1 and 950 parts of mixed particle sizes of AlN was added as filler, the thermal conductivities of the prepared silicone paste were 4.22 and 4.36 W/(m∙K), while their thermal resistances were 4.99 and 4.01 K∙cm^2^/W, respectively. These results indicated that TMSM-PDES-TMOS exhibited a better dispersion effect on mixed AlN fillers. Although it might be possible to obtain silicone paste with high thermal conductivity using TMSM-PDES-TMOS as the surface modifying agent for AlN fillers, its high price and cost prevented its large-scale application in TIMs.

### 3.5. Effect of Polysiloxane Structure on the Surface and Interface Morphology of Composite Materials

**Figure 4:**
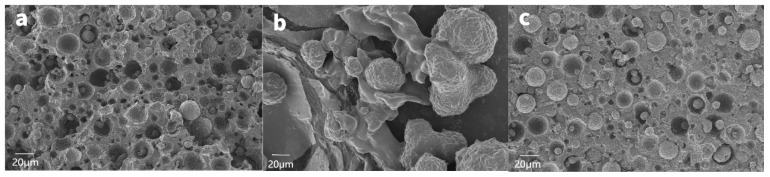
SEM images of AlN powder-filled silicone rubber in which AlN was treated with different polysiloxanes ((**a**) TMSM-PDMS-TMOS; (**b**) TMSM-PTFPMS-TMOS; (**c**) TMSM-PDES-TMOS).

To reveal the effect of polysiloxane structures on the surface of AlN fillers and the dispersion behaviors of treated AlN in the polysiloxane matrix, cured thermally conductive silicone rubbers were prepared (see Section 2.3.2 for the preparation process) and were subjected to scanning electron microscopy as shown in Figure 4a–c.

It can be seen from Figure 4 that when polysiloxanes with different side chain structures were functionalized as the surface treatment agents for AlN particles, the dispersion of AlN fillers in the composites varied greatly. In Figure 4a, the AlN filler was uniformly dispersed in the composites. In Figure 4b, a large amount of agglomeration of the AlN fillers was observed. The higher the quantity of powder, the more serious the agglomeration. In Figure 4c, no obvious agglomeration was observed and the dispersion of AlN fillers seemed better than that observed in Figure 4b. However, some sporadic filler agglomeration could still be observed when compared with Figure 4a. The SEM results showed that AlN fillers were dispersed in different ways in the polysiloxane matrix (terminal vinyl-containing silicone fluid) after the AlN fillers were treated with different polysiloxane surface treatments with different side chain structures, which led to significant differences in the thermal conductivity of the composites produced. When TMSM-PTFPMS-TMOS was used as the AlN filler surface treatment agent, the dispersion of the AlN filler in the polymer matrix was the poorest and obvious agglomeration appeared in composites, resulting in failure to form a continuous thermal conduction path. The reason is briefly discussed in Section 3.4.2. Obviously, this kind of polysiloxane was not suitable to be functionalized as the surface treatment for AlN fillers in the preparation of TIMs with higher thermal conductivity. When TMSM-PDMS-TMOS was used as the AlN filler treatment agent, SEM showed that the treated AlN filler was uniformly dispersed in the polymer matrix, and an effective heat conduction path was formed inside the composite material. The thermal conductivity of the composite reached 4.81 W/(m∙K) with a thermal resistance of 4.37 K∙cm^2^/W when 1200 parts of AlN fillers with mixed particle sizes was added.

When TMSM-PDES-TMOS was used as the AlN filler treatment agent, SEM showed that the dispersion of AlN fillers in the polymer matrix after treatment was greatly improved relative to that obtained from TMSM-PTFPMS-TMOS. Nevertheless, a small extent of agglomeration could still be observed in the SEM picture (Figure 4c). With the addition of 950 parts of mixed-size AlN fillers and treatment in situ with TMSM-PDES-TMOS, the thermal conductivity of the composite reached a maximum value of 4.36 W/(m·K), together with a thermal resistance of 4.01 K∙cm^2^/W.

Therefore, TMSM-PDMS-TMOS was the most effective for the surface treatment of AlN filler among the three novel polysiloxanes investigated in this study, which might be related to the smallest steric hindrance of the Si–CH_3_ group in the side chain of polysiloxane under investigation. On the other hand, Si–CH_3_ also had the least influence on the Si–O–Si bond angle and would not affect the helical structure of the Si–O–Si bond or the flexibility of the polysiloxane segment. Consequently, taking TMSM-PDMS-TMOS as the surface treatment agent for AlN fillers could effectively improve the dispersion performance of AlN fillers in a polysiloxane matrix, and thus, it was expected to prepare TIMs with higher thermal conductivity.

### 3.6. Possible Mechanism of Surface Treatment of AlN Fillers

To further explore the reason why different structures of monotrimethoxylsilylethyl-terminated polysiloxane fluids had different influences on the dispersion of treated AlN fillers in the terminal vinyl-containing silicone fluid matrix, FT-IR spectra of untreated AlN filler and those of treated AlN fillers with TMSM-PDMS-TMOS and TMSM-PTFPMS-TMOS were recorded and compared.

**Figure 5:**
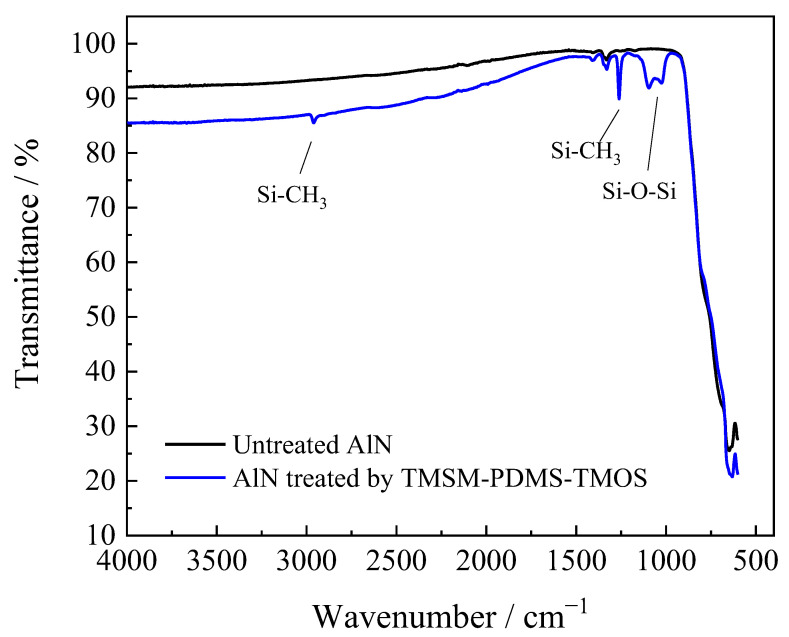
Comparison of FT-IR spectra of untreated AlN and AlN treated by TMSM-PDMS-TMOS.

In a 100 mL mixing cup, 0.5 g of TMSM-PDMS-TMOS, 10 g of AlN (1 μm) filler and 5 mL acetone were added. The mixing cup was then put into a noninterventional material homogenizer for stirring and defoaming treatment (1800 rpm, −99.5 kPa) for 3 min. The treated AlN filler was taken out and heated at 50 °C for 1 h to completely volatilize the acetone. The FT-IR spectra (KBr tablet method) of untreated AlN filler and those of AlN fillers treated with TMSM-PDMS-TMOS were recorded and are shown in Figure 5. It can be seen from Figure 5 that after treating with TMSM-PDMS-TMOS, an obvious Si-O-Si asymmetric vibration absorption peak appeared between 1000 and 1100 cm^−1^. Meanwhile, a C-H stretching vibration absorption peak appeared at 2964 cm^−1^ and Si-CH_3_ stretching vibration absorption peak appeared at 1254 cm^−1^, indicating that the organic silicone component that resulted from TMSM-PDMS-TMOS was successfully introduced onto the surface of AlN filler.

**Figure 6:**
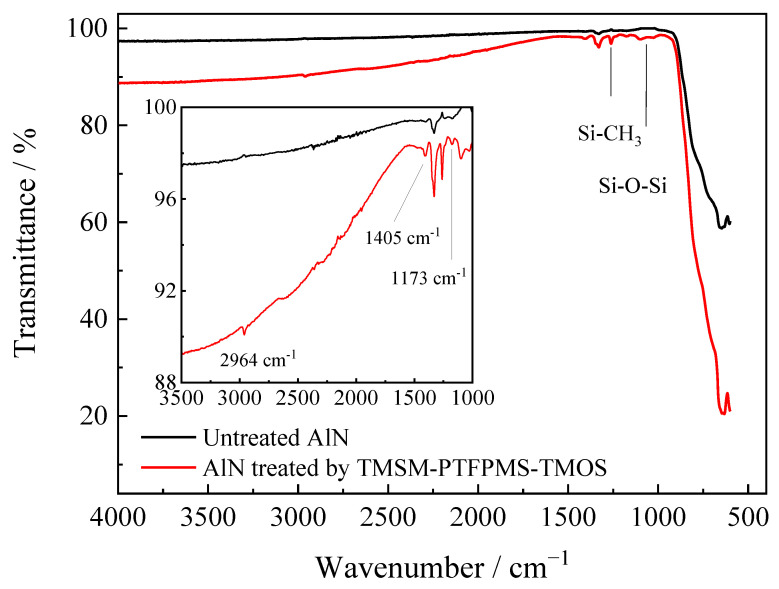
Comparison of FT-IR spectra of untreated AlN and AlN treated by TMSM-PTFPMS-TMOS.

When TMSM-PDMS-TMOS was substituted by TMSM-PTFPMS-TMOS with a viscosity of 10 mPa.s at 25 °C and functioned as the surface treatment agent for AlN fillers, the influence of TMSM-PTFPMS-TMOS on the surface of AlN filler was also investigated. The FT-IR spectra (KBr tablet method) of untreated AlN filler and that of AlN fillers treated with TMSM-PTFPMS-TMOS were recorded and are shown in Figure 6.

It can be seen clearly from Figure 6 and its inset enlarged view between 1000 and 3500 cm^−1^ that a weak Si-O-Si bond formed (between 1000 and 1100 cm^−1^) in TMSM-PTFPMS-TMOS-treated AlN filler, while Si-CH_3_ could also be observed in the FT-IR spectra (2964 and 1260 cm^−1^). In the inset enlarged view, 1405 cm^−1^ and 1173 cm^−1^ were attributed to the vibration absorption peak of –CH_2_-CH_2_- and the -CF_3_ group in the trifluoropropyl group, respectively. The FT-IR spectra proved that TMSM-PTFPMS-TMOS had successfully reacted with the hydroxyl groups on the surface of AlN, but the concentration of Si-O-Si bonds formed was not as high as that formed between TMSM-PDMS-TMOS and AlN fillers.

Because the surface treatment effect of TMSM-PTFPMS-TMOS on the surface of AlN filler was inferior to that of TMSM-PDMS-TMOS, the dispersion of AlN particles modified with TMSM-PTFPMS-TMOS in terminal vinyl-containing silicone fluid was poor. In Figure 4b, obvious particle agglomeration could be observed and AlN fillers with different sizes were separated, which prevented the formation of effective heat transfer pathways. For TMSM-PDMS-TMOS, its SEM image (Figure 4a) exhibited a completely different result. In Figure 4a, not only was no obvious particle agglomeration observed, but the gaps between AlN filler particles of different particle sizes were small, which helped the formation of thermally conductive pathways and improved the thermal conductivity of TIMs.

**Figure 7:**
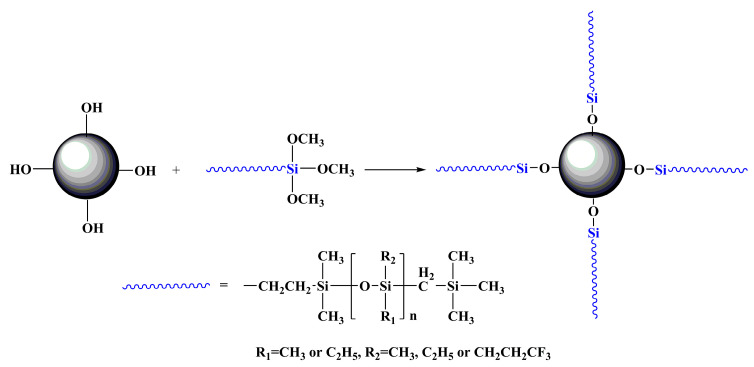
Surface treatment mechanisms of AlN fillers treated by monotrimethoxylsilylethyl-terminated polysiloxanefluids.

Based on the SEM analysis and comparisons of FT-IR spectra of untreated and treated AlN fillers, a surface treatment mechanism of AlN fillers treated by monotrimethoxylsilylethyl-terminated polysiloxane fluids was presented and is illustratively shown in Figure 7.

**Figure 8:**
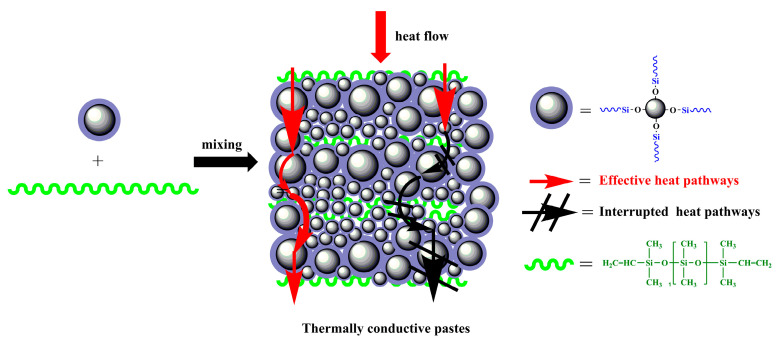
Schematic diagram of heat transfer in modified AlN-filled thermally conductive paste.

Benefiting from the grafted monotrimethylsilyl-terminated polysiloxane on AlN fillers through the condensation reaction between the hydroxyl group in AlN fillers and the terminal trimethoxy group in monotrimethoxylsilylethyl-terminated polysiloxane fluids as shown in Figure 7, a good compatibility between AlN fillers and terminal vinyl-containing silicone fluid could be obtained. Because of the uniform dispersion of AlN fillers treated with TMSM-PDMS-TMOS in terminal vinyl-containing silicone fluid, the filling load of AlN fillers in the silicone oil matrix could be greatly enhanced. Under such circumstances, more thermal conductivity pathways in TIMs could be formed as illustrated in Figure 8; therefore, monotrimethoxylsilylethyl-terminated polysiloxane fluids were crucial to develop TIMs with high thermal conductivity and good fluidity.

## 4. Conclusions

D_3_, D_3_^Et^ and D_3_^F^ could undergo nonequilibrium ring-opening polymerization initiated by TMSiMLi. When using DMCS as a chain terminator, *α*-trimethylsilylmethyl-*ω*-dimethylsilyl-terminated siloxane oligomers could be obtained. Hydrosilylation of *α*-trimethylsilylmethyl-*ω*-dimethylsilyl-terminated siloxane oligomers with vinyltrimethoxysilane could give *α*-trimethylsilylmethyl-*ω*-trimethoxysilylethyl-terminated siloxane oligomers. Among the three six-membered cyclotrisiloxanes investigated, the ring-opening yield of D_3_^Et^ was the lowest.

Using *α*-trimethylsilylmethyl-*ω*-trimethoxysilylethyl-terminated siloxane oligomer as the in situ surface treatment agent for AlN fillers, modified AlN filler-filled silicone paste and silicone rubber could be produced. When TMSM-PTFPMS-TMOS was used as the surface treatment agent for AlN fillers with mixed particle sizes, in the investigated range, the thermal conductivity of the silicone paste prepared by these modified AlN fillers showed a decreased trend with the increase in the filler content, while its thermal resistance exhibited an increased trend. The SEM images showed that there was an obvious phase separation between the AlN filler and the polymer matrix, resulting in a decrease in the thermal conductivity of the composite materials.

Although the thermal conductivity of TMSM-PDES-TMOS-treated AlN filler-filled paste was slightly higher than that of TMSM-PDMS-TMOS-treated AlN filler, considering the high price of D_3_^Et^ and the lowest yield of ring-opening polymerization, TMSM-PDMS-TMOS was more cost-effective as a surface treatment agent for AlN fillers. When mixing TMSM-PDMS-TMOS with terminal vinyl-containing silicone fluid in a mass ratio of 1:2 and setting the total mass of these two polysiloxanes as a reference (100 parts), 1200 parts AlN fillers with mixed particle sizes (AlN particles with particle sizes of 50 μm, 20 μm and 1 μm were added in a mass ratio of 9:5:3) filled them, and thus, a silicone paste with a thermal conductivity of 4.81 W/(m∙K) and good fluidity was obtained. Therefore, TMSM-PDMS-TMOS was expected to improve the surface properties of AlN greatly and was beneficial to the preparation of composite materials with higher thermal conductivity.

## Figures and Tables

**Table 1:** The molecular characteristics of all the samples.

Sample ID	*M*n (g/mol)	*M*w (g/mol)	PDI	*M* ^b^ _NMR_ (g/mol)
TMSM-PDMS-DMS	1611	2048	1.27	908
TMSM-PDMS-TMOS	ND ^a^	ND	ND	1056
TMSM-PTFPMS-DMS	857	1051	1.23	1628
TMSM-PTFPMS-TMOS	ND	ND	ND	1776
TMSM-PDES-DMS	718	879	1.22	782
TMSM-PDES-TMOS	ND	ND	ND	930

Note: ^a^ ND = not detected; ^b^ Molecular weight calculated from ^1^H NMR spectra.

**Table 2:** Effect of reaction time on the yield of TMSM-PDMS-DMS.

Entry	n*	*m*_D3_/g	*V*_TMSiMLi_/mL	*V*_THF_/mL	*m*_DMCS_/g	*t*/h	*T*/°C	Yield/%
A1	10	10.00	20.00	30.00	1.75	15	40	59.0
A2	10	10.06	20.00	30.00	1.75	13	40	50.1
A3	10	10.07	20.00	30.00	1.75	9	40	60.1
A4	10	10.00	20.00	30.00	1.75	6	40	80.0

Note: n* denotes the designed degree of polymerization.

**Table 3:** Effect of solvent types on the yield of TMSM-PTFPMS-DMS.

Entry	n*	*m*_D3F_/g	*V*_TMSiMLi_/mL	*V*_1_/mL	*m*_DMCS_/g	Solvent	*t*/h	*T*/°C	Yield/%
B1	10	10.02	12.00	10.00	1.25	THF	8	40	50.1
B2	10	10.02	12.00	10.00	1.25	DMSO	8	40	18.0
B3	10	10.02	15.00	10.00	1.25	NMP	8	40	42.0

Note: n* denotes the design degree of polymerization and *V*_1_ is the volume of different solvents. All the solvents used were ultra-dry solvents.

## Data Availability

Structural characterization of prepared monotrimethoxylsilylethyl-terminated polysiloxane fluids can be found in the Supplementary Information of this manuscript.

## Supplementary Materials

The following supporting information can be downloaded at: https://www.mdpi.com/article/10.3390/polym15163334/s1, Figure S1: ^1^H NMR spectrum of TMSM-PDMS-DMS; Figure S2: ^29^Si NMR spectrum of TMSM-PDMS-DMS; Figure S3: ^13^C NMR spectrum of TMSM-PDMS-DMS; Figure S4: FT-IR spectrum of TMSM-PDMS-DMS; Figure S5: GPC curve of TMSM-PDMS-DMS; Figure S6: TGA and DTG curves of TMSM-PDMS-DMS; Figure S7: ^1^H NMR spectra of TMSM-PDMS-TMOS; Figure S8: ^29^Si NMR spectra of TMSM-PDMS-TMOS; Figure S9: ^13^C NMR spectra of TMSM-PDMS-TMOS; Figure S10: FT-IR spectra of TMSM-PDMS-TMOS; Figure S11: TGA and DTG curves of TMSM-PDMS-TMOS; Figure S12: ^1^H NMR spectra of TMSM-PTFPMS-DMS; Figure S13: ^13^C NMR spectra of TMSM-PTFPMS-DMS; Figure S14: ^29^Si NMR spectra of TMSM-PTFPMS-DMS; Figure S15: FT-IR spectra of TMSM-PTFPMS-DMS; Figure S16: TGA and DTG curves of TMSM-PTFPMS-DMS; Figure S17: DSC curve of TMSM-PTFPMS-DMS; Figure S18: GPC curve of TMSM-PTFPMS-DMS; Figure S19: ^1^H NMR spectra of TMSM-PTFPMS-TMOS; Figure S20: ^13^C NMR spectra of TMSM-PTFPMS-TMOS; Figure S21: ^29^Si NMR spectra of TMSM-PTFPMS-TMOS; Figure S22: ^19^F NMR spectra of TMSM-PTFPMS-TMOS; Figure S23: FT-IR spectra of TMSM-PTFPMS-TMOS; Figure S24: TGA and DTG curves of TMSM-PTFPMS-TMOS; Figure S25: ^1^H NMR spectra of TMSM-PDES-DMS; Figure S26: ^13^C NMR spectra of TMSM-PDES-DMS; Figure S27: ^29^Si NMR spectra of TMSM-PDES-DMS; Figure S28: FT-IR spectra of TMSM-PDES-DMS; Figure S29: TGA and DTG curves of TMSM-PDES-DMS; Figure S30: DSC curve of TMSM-PDES-DMS; Figure S31: GPC curve of TMSM- PDES -DMS; Figure S32: ^1^H NMR spectra of TMSM-PDES-TMOS; Figure S33: ^13^C NMR spectra of TMSM-PDES-TMOS; Figure S34: ^29^Si NMR spectra of TMSM-PDES-TMOS; Figure S35: FT-IR spectra of TMSM-PDES-TMOS; Figure S36: TGA and DTG curves of TMSM-PDES-TMOS.
