# Supplementary material for: Preparation of Monotrimethoxylsilylethyl-Terminated Polysiloxane Fluids and Their Application in Thermal Interface Materials
Source: Polymers (Basel). 2023 Aug 8;15(16):3334. doi: 10.3390/polym15163334 (PMC10460069; doi:10.3390/polym15163334)
Supplement: Supplementary file 1 [file polymers-15-03334-s001.zip › polymers-2501928-supplementary.pdf]

polymers

Supporting Information

Structure Characterization and Thermal Performance of Synthesized Silicone Fluids and Their Precursors

1. TMSM-PDMS-TMOS

1.1. TMSM-PDMS-DMS

The ^{1}H NMR spectrum of the prepared TMSM-PDMS-DMS was shown in Figure S1 in Supporting Information. It could be observed that the chemical shift in the neighborhood of $\delta = -0.1$ ppm could be attributed to the two protons in the methylene group between two Si atoms, namely, Si–C**H**$_2$–Si. The chemical shift between $\delta = 0$ and 0.5 ppm represented the proton peak on all methyl groups (Si–C**H**$_3$) attached to the side chain of a silicon atom and their number of protons shown in the illustrative structure of the polymer in the insert picture of Figure S1 was 6*n* + 15. The chemical shift at about $\delta = 1.6$ ppm was the hydroxyl peak of the water peak, which came from impurities in the sample or moisture introduced during the testing process. The chemical shift at about $\delta = 4.7$ ppm was attributed to the characteristic peak of protons in the Si–**H** bond. The ^{1}H NMR spectra indicated that the chemical environment of each proton was consistent with the structure of the synthesized TMSM-PDMS-DMS, the value of *n* could be calculated from the area integration of Si–C**H**$_3$ relative to that of Si–C**H**$_2$–Si and the molecular weight of the polymer M_{NMR} could also be calculated.

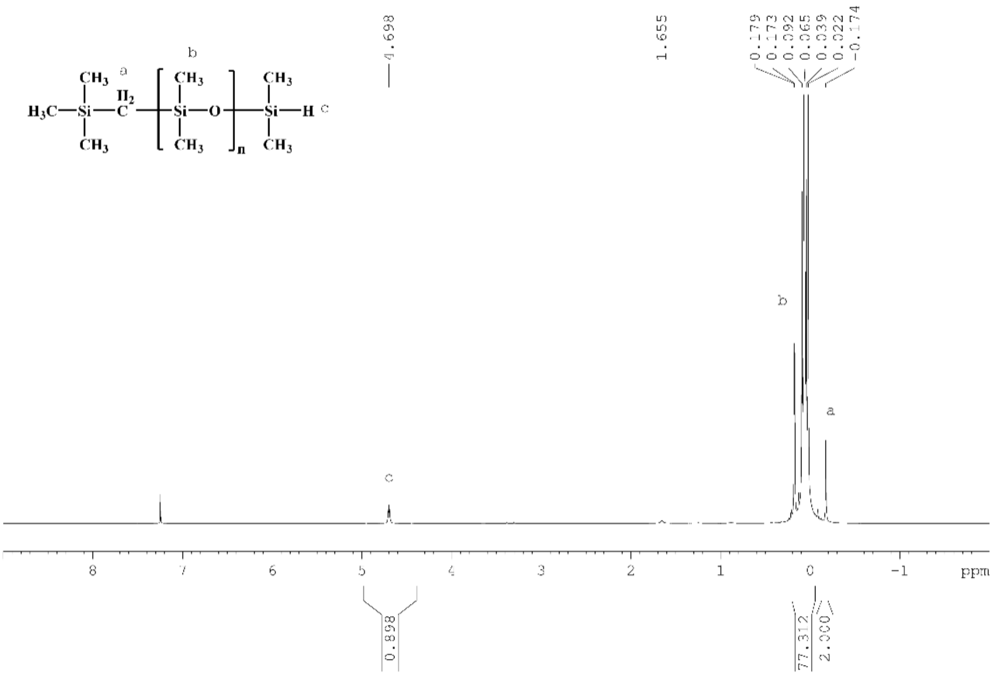

Figure S1. ^{1}H NMR spectrum of TMSM-PDMS-DMS.

The ^{29}Si NMR spectrum of the prepared TMSM-PDMS-DMS was shown in Figure S2 in Supporting Information. It could be seen from Figure S2 that there were four kinds of silicon atoms with different chemical shifts in the ^{29}Si NMR spectrum of the tested sample, among which the chemical shift at $\delta = 6.5$ ppm was the characteristic peak of a Si atom

connected with a methyl group (***Si***–CH_3). The chemical shift at δ= –7 ppm was the characteristic peak of a Si atom connected with a hydrogen atom (***Si***–H). The chemical shift at δ= –0.7 ppm was the characteristic peak of a Si atom connected with a methylene group (***Si***–CH_2–) and the chemical shift at δ= –21 ppm was the characteristic peak of a Si atom in the D segment (–O–***Si***$(CH_3)_2$–O–). The analysis results of ^{29}Si NMR of the sample showed that the chemical environment of the silicon atom in the tested sample was consistent with that of TMSM-PDMS-DMS.

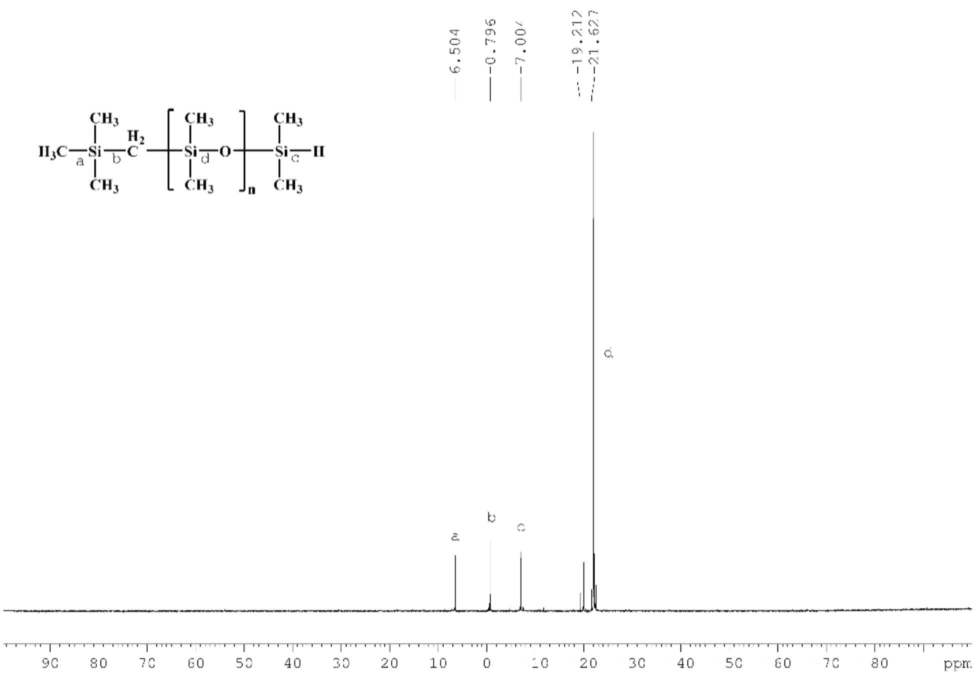

Figure S2. ^{29}Si NMR spectrum of TMSM-PDMS-DMS.

The ^{13}C NMR spectrum of the prepared TMSM-PDMS-DMS was shown in Figure S3 in Supporting Information. It could be seen from Figure S3 that the chemical shift at δ= 0 ppm was the characteristic peak of the carbon atom in the repeating unit of –O–Si($\underline{C}H_3)_2$–O–. The chemical shift at δ= 5 ppm was the characteristic peak of the carbon atom in Si–$\underline{C}H_2$– and the chemical shift at δ= 2 ppm was the characteristic peak of carbon atoms in the Si–$\underline{C}H_3$ group located at both ends of the polymer.

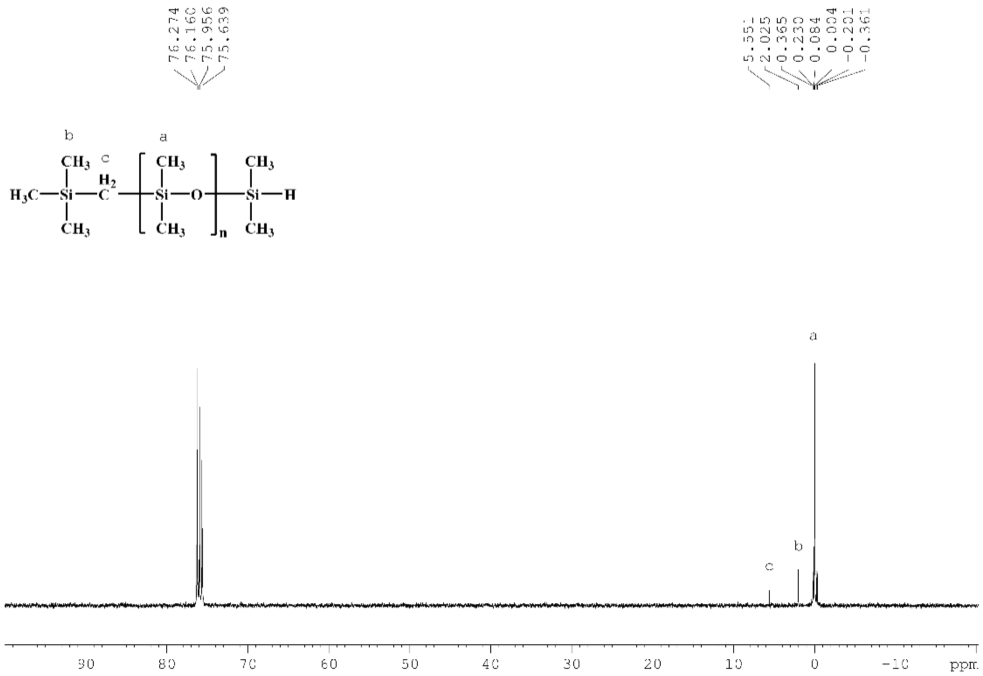

Figure S3. ^{13}C NMR spectrum of TMSM-PDMS-DMS.

From the infrared spectrum shown in Figure S4 in Supporting Information, it could be found that the broad and strong absorption band ranging from 1130 cm^{-1} to 1000 cm^{-1} was the asymmetric vibration absorption peak of Si–O–Si, and the band at 2964 cm^{-1} was the C–H stretching vibration absorption peak. Peaks at 1259 cm^{-1} and 787 cm^{-1} were Si–CH_3 stretching vibration absorption peaks in siloxane and the peak at 2100 cm^{-1} was the Si–H characteristic peak.

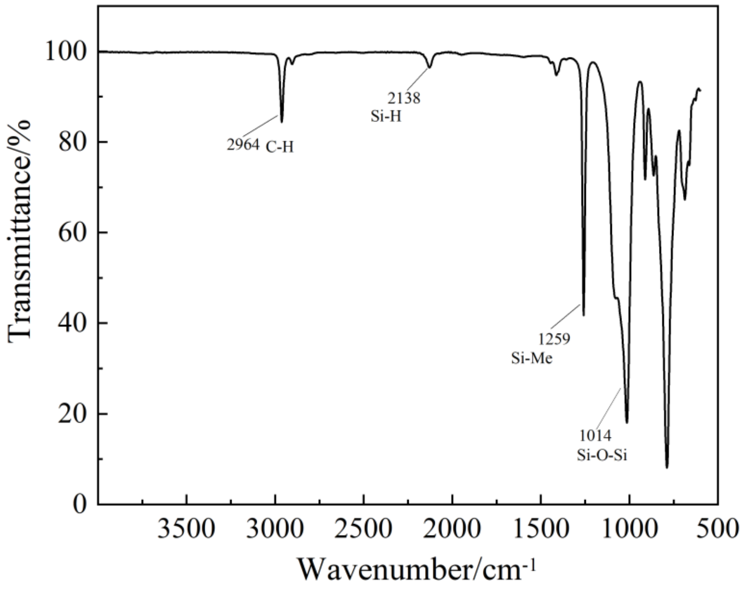

Figure S4. FT-IR spectrum of TMSM-PDMS-DMS.

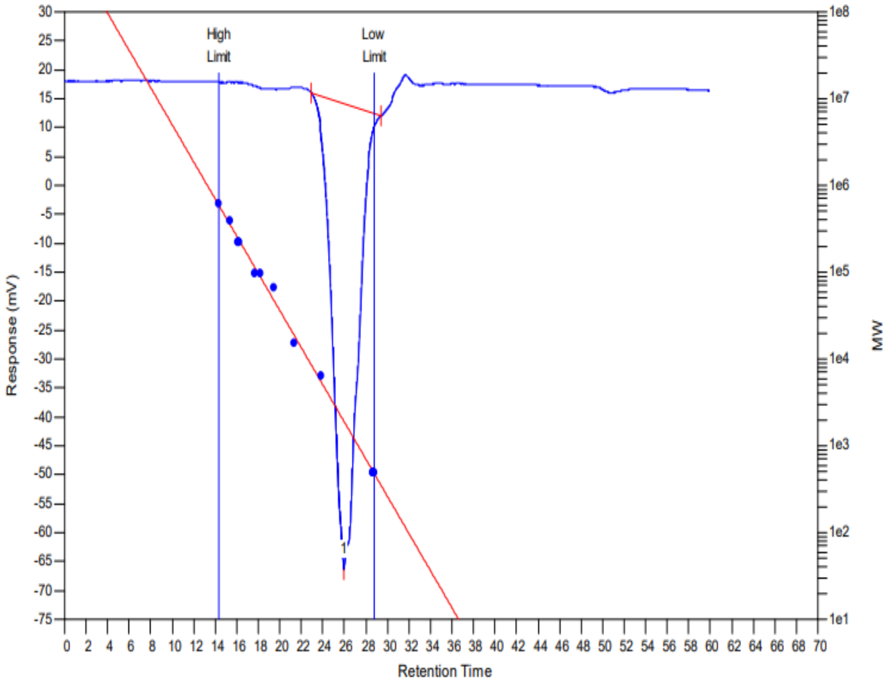

Figure S5. GPC curve of TMSM-PDMS-DMS.

With PDMS samples of standard molecular weights as the reference substance (490, 6200, 15000, 34100, 64800, 95800, 219400, 384500 and 610000 g/mol, respectively) and toluene as the mobile phase, the sample was tested using GPC. The measured weight average molar mass (*M*w) of the sample was 2048 g/mol, the number average molar mass (*M*n) was 1611 g/mol and the polydispersity index (PDI) was 1.27. It could also be observed from the GPC curve (Figure S5 in Supporting Information) that there was only one peak in the GPC curve, which obviously exhibited the characteristics of a normal distribution of high molecular weight polymer and proves the sample was a single component polymer. TLC was used to further confirm that the product was substantially a single component. Because the PDI value was small (1.27) and the peak in the GPC curve was narrow and sharp, it could be concluded that the prepared polymer had a concentrated molecular weight distribution and possessed the characteristics of a narrow molecular weight distribution polymer.

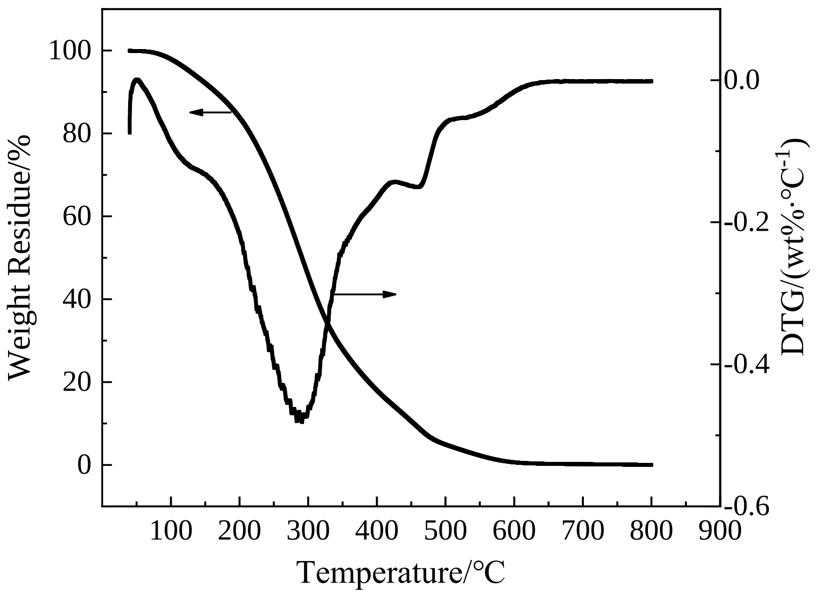

Figure S6. TGA and DTG curves of TMSM-PDMS-DMS.

From the TGA and DTG curves shown in Figure S6 in Supporting Information, it could be observed that the temperatures at weight loss 5% ($T_{5\%}$), 10% ($T_{10\%}$) and the temperature at which the sample reached the fastest thermal degradation rate (T_{max}) were 127.6, 165.5 and 300.1 °C, respectively. One could find from the DTG curve that as the temperature rose, the sample showed a small amount of thermal degradation behavior at around 120 °C, which might be caused by the sublimation of a small amount of D_3 remaining in the sample. When the temperature rose to about 300 °C, the thermal degradation rate of the polymer reached the maximum value with a maximum thermal degradation rate of −0.47 wt%/°C. This might be caused by the depolymerization of the polymer at higher temperatures and the formation of volatile cyclosiloxanes. When the temperature continued to rise to about 470 °C and 560 °C, a small thermal degradation peak and a plateau peak appeared in the thermal degradation curve of the polymer and their thermal degradation rates were −0.16 wt%/°C and −0.06 wt%/°C, respectively. These two degradations might originate from the mass loss caused by small molecules released during the conversion of Si–CH_3 bonded to Si atoms or the Si–C bond in the capping group to the SiC ceramic phase structure at higher temperatures. The TGA and DTG curves of other prepared polymers showed similar shapes, indicating that the degradation of these polymers followed a similar reaction mechanism, but their $T_{5\%}$, $T_{10\%}$ and T_{max} values differed with the molecular structure.

1.2. TMSM-PDMS-TMOS

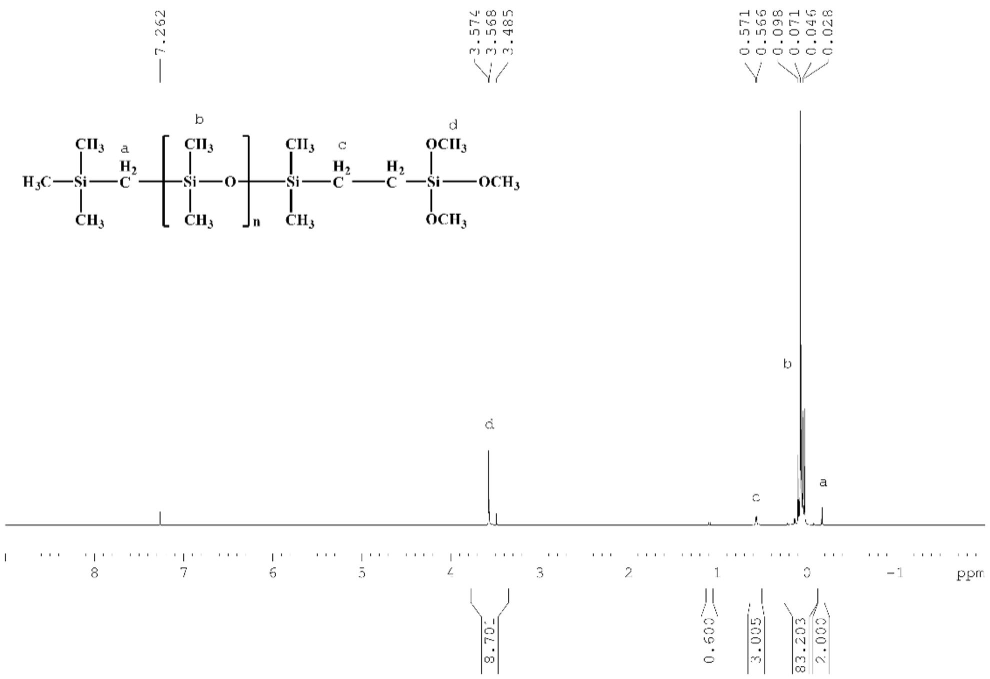

Figure S7. ^{1}H NMR spectra of TMSM-PDMS-TMOS.

The ^{1}H NMR spectrum of the prepared TMSM-PDMS-TMOS was shown in Figure S7 in Supporting Information. It could be observed that the chemical shift in the neighborhood of $\delta = -0.1$ ppm could be attributed to the two protons in the methylene group between two Si atoms, namely, Si–C$\underline{\boldsymbol{H}}_2$–Si. The chemical shift between $\delta = 0$ and 0.5 ppm represented the proton peak on all methyl groups (Si–C$\underline{\boldsymbol{H}}_3$) attached to the side chain of the silicon atom and their number of protons as shown in the illustrative structure of the polymer in the insert picture of Figure S7 was $6n + 15$. The chemical shift at $\delta = 0.57$ ppm was attributed to the characteristic peak of the addition product obtained following Markovnikov's rule. There was a characteristic peak of the addition product of the reverse Markovnikov rule at the chemical shift $\delta = 1.2$ ppm. The chemical shift at $\delta = 3.5$ ppm was attributed to the proton of trimethoxy (–Si–OC$\underline{\boldsymbol{H}}_3$) obtained by the hydrosilylation reaction. The value of n in the structural formula and molecular weight M_{NMR} could be calculated from the area integration of Si–C$\underline{\boldsymbol{H}}_3$ relative to that of Si–C$\underline{\boldsymbol{H}}_2$–Si.

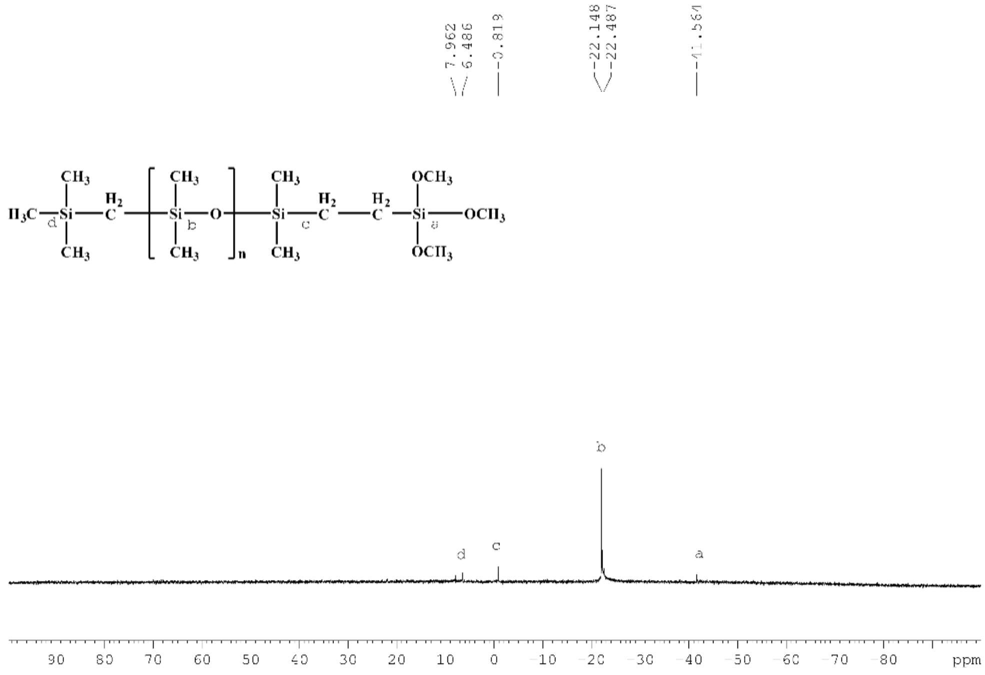

Figure S8. ^{29}Si NMR spectra of TMSM-PDMS-TMOS.

The ^{29}Si NMR spectrum of the prepared TMSM-PDMS-TMOS was shown in Figure S8 in Supporting Information. The signal at δ= –0.7 ppm was the chemical shift of the silicon atom (–***Si***–CH_2–) connected to the methylene group. The peak around δ= –21 ppm was attributed to the silicon atom connected to the methyl group in the D segment (–O–***Si***(CH_3)$_2$–O–). Compared with Figure S2, it can be clearly seen that the chemical shift of the silicon atom connected to the hydrogen atom (***Si***–H) at δ= −7 ppm disappeared, and at the same time, a chemical shift of the silicon atom attached to the methoxy group (–***Si***–OCH_3) appeared at δ= −41 ppm, indicating that the Si–H functional group had been converted to –Si–O–CH_3.

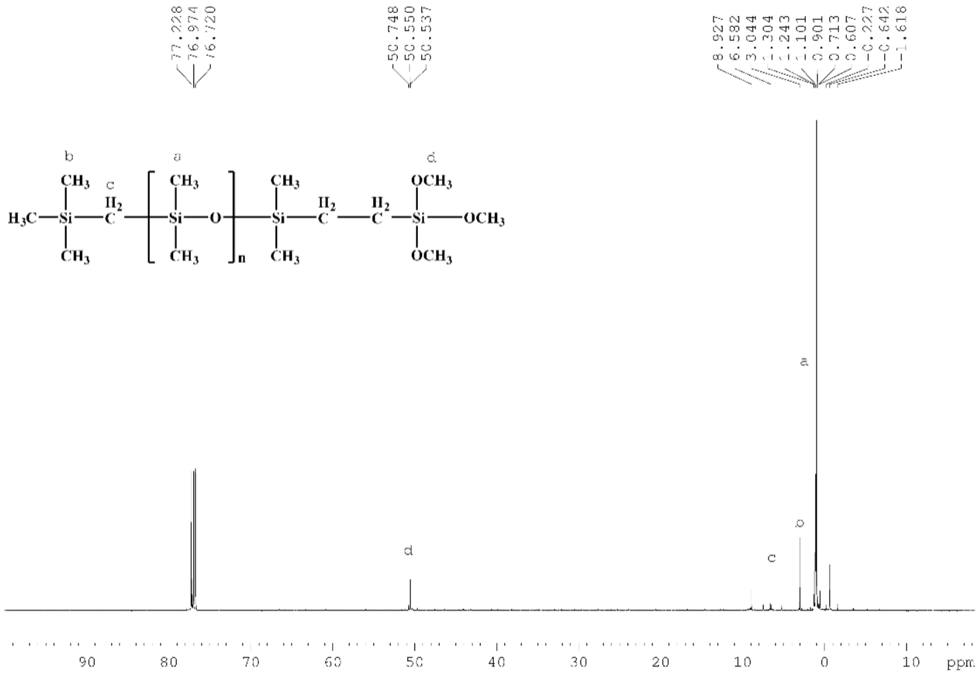

Figure S9. ^{13}C NMR spectra of TMSM-PDMS-TMOS.

The ^{13}C NMR spectrum of the prepared TMSM-PDMS-TMOS was shown in Figure S9 in Supporting Information. It could be observed from Figure S9 that the chemical shift near δ= 0 ppm was attributed to the carbon atom on the side chain methyl group (Si–$\underline{C}H_3$) connected to the Si atom in the PDMS. The chemical shift at δ= 5 ppm was attributed to the carbon atom in the methylene group (Si–$\underline{C}H_2$) connected to the Si atom. The chemical shift at δ= 2 ppm was attributed to the carbon atom in the methyl group attached to the Si atom (Si–$\underline{C}H_3$) and located at the end of the polymer. The chemical shift at δ= 50 ppm was attributed to the carbon atom in the methoxy group (Si–O$\underline{C}H_3$) connected to the Si atom and located at the end of the polymer.

It could be seen from Figure S10 in Supporting Information that the broad and strong absorption band between 1130 cm^{-1} and 1000 cm^{-1} was the asymmetric stretching vibration absorption peak of Si–O–Si. The C–H stretching vibration absorption peak was located at 2961 cm^{-1}. The wavenumbers 1258 cm^{-1} and 787 cm^{-1} were the Si–CH_3 stretching vibration absorption peaks in siloxane and 2840 cm^{-1} was the characteristic peak of the Si–OCH_3 group.

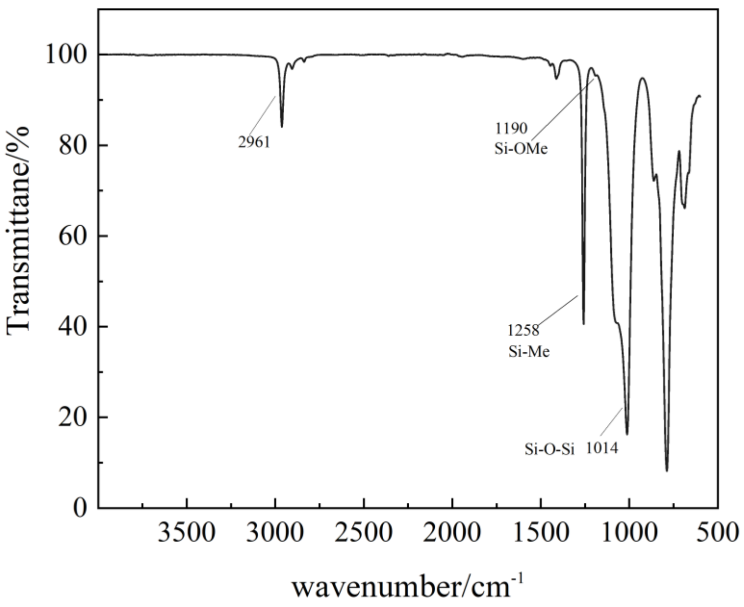

Figure S10. FT-IR spectra of TMSM-PDMS-TMOS.

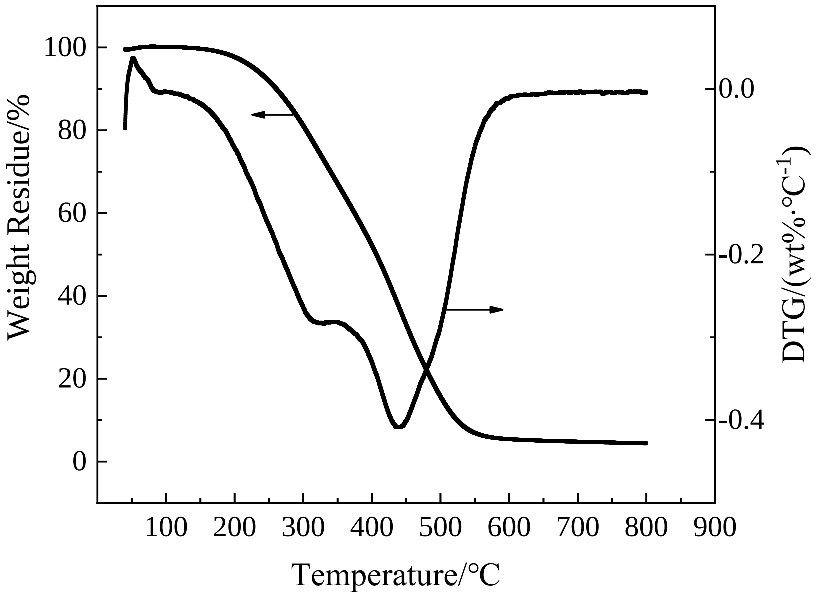

Figure S11. TGA and DTG curves of TMSM-PDMS-TMOS.

The temperatures at $T_{5\%}$, $T_{10\%}$ and T_{max} of TMSM-PTFPMS-TMOS determined from the TGA and DTG curves (Figure S11 in Supporting Information) were 228.4, 260.8 and 430.2 °C, respectively. As the temperature increased, the sample showed a small amount of thermal degradation behavior at about 100 °C. When the temperature rose to about 430 °C, a maximum thermal degradation was observed, and its maximum thermal degradation rate was −0.45 wt%/°C.

2. TMSM-PTFPMS-TMOS

2.1. TMSM-PTFPMS-DMS

The ^1H NMR, ^{13}C NMR, ^{29}Si NMR and FT-IR spectra of TMSM-PTFPMS-DMS were shown in Figures S12 to S15 in the Supporting Information and their characterization information was listed below.

^1H NMR (400 MHz, Chloroform-*d*): δ = 4.71 ppm (s, *J* = 2.9 Hz, 1H), 2.03 ppm (ddt, *J* = 18.4, 13.5, 9.6 Hz, 26H), 1.02–0.59 ppm (m, 24H), 0.38 –−0.08 ppm (m, 50H), −0.14 (s, 2H). ^{13}C NMR (126 MHz, Chloroform-*d*): δ = 132.55–122.56 ppm (m), 29.94–26.06 ppm (m), 9.58–7.35 ppm (m), 2.05–−2.72 ppm (m), −3.61–−4.96 ppm (m). ^{29}Si NMR (99 MHz, Chloroform-*d*): δ = 11.98–6.19 ppm (m), −0.71, −3.76–−6.90 ppm (m), −7.22–−25.03 ppm (m). ^{19}F NMR (471 MHz, Chloroform-*d*): δ = −68.59–−69.01 ppm (m). FT-IR: 1130–1100 cm^{-1} (Si–O–Si), 2961 cm^{-1} (C–H), 1261 and 787 cm^{-1} (Si–CH_3), 1210 cm^{-1} (CF_3), 1369 cm^{-1} (CH_2CH_2), 2134 cm^{-1} (Si–H).

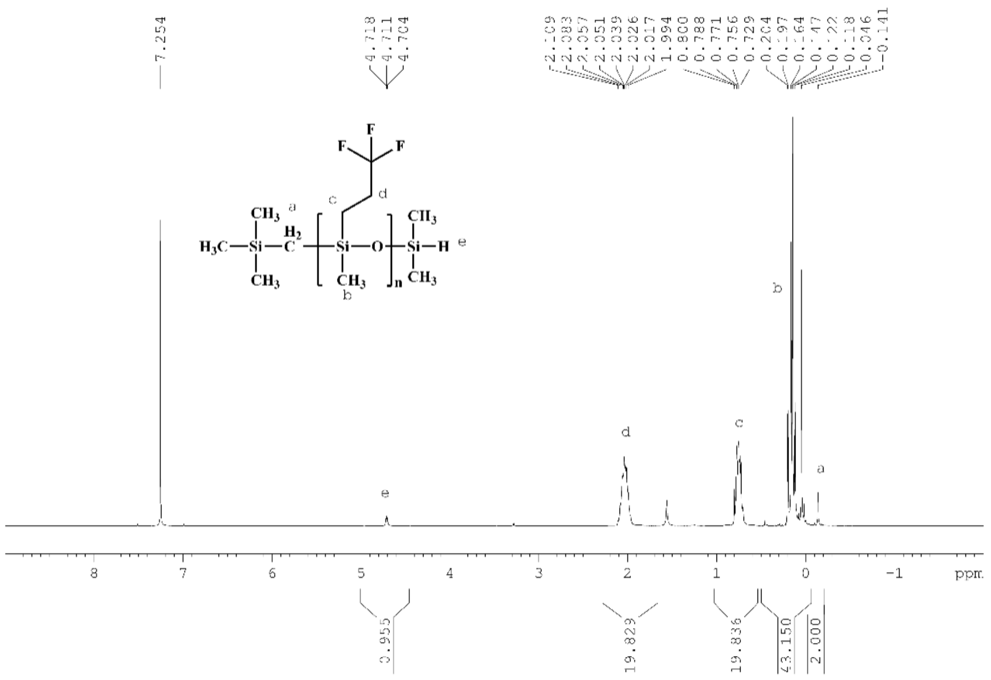

Figure S12. ^1H NMR spectra of TMSM-PTFPMS-DMS.

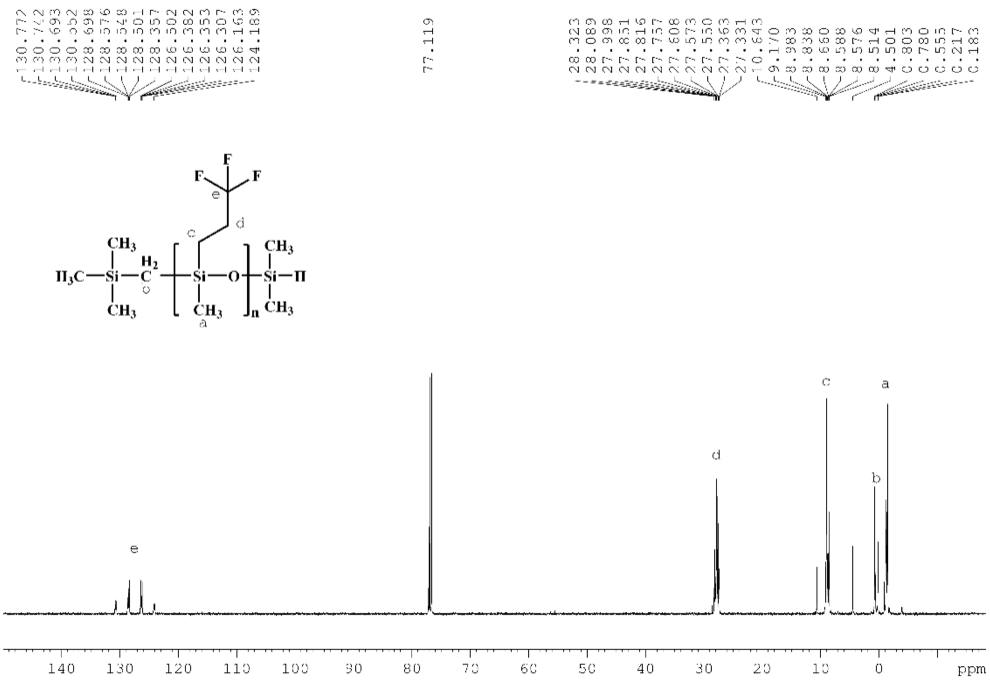

Figure S13. ^{13}C NMR spectra of TMSM-PTFPMS-DMS.

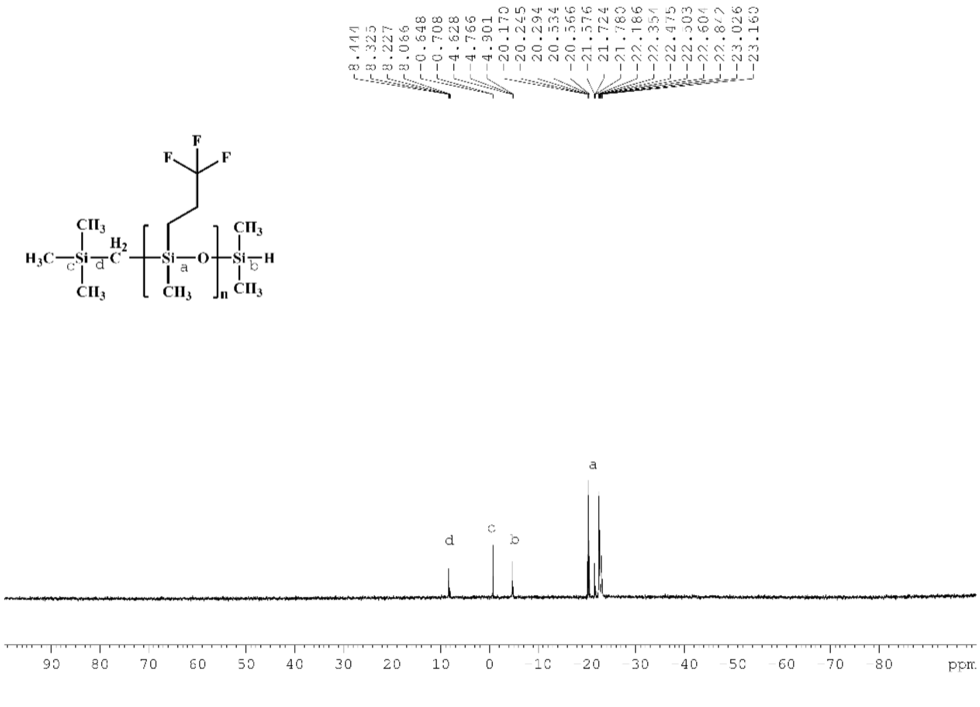

Figure S14. ^{29}Si NMR spectra of TMSM-PTFPMS-DMS.

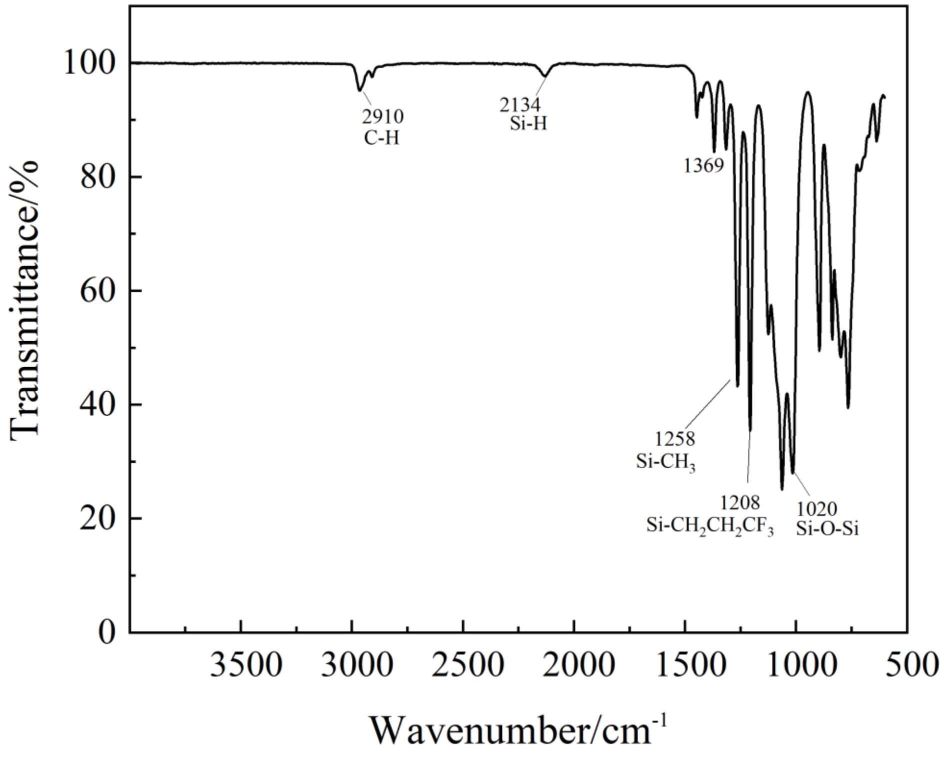

Figure S15. FT-IR spectra of TMSM-PTFPMS-DMS.

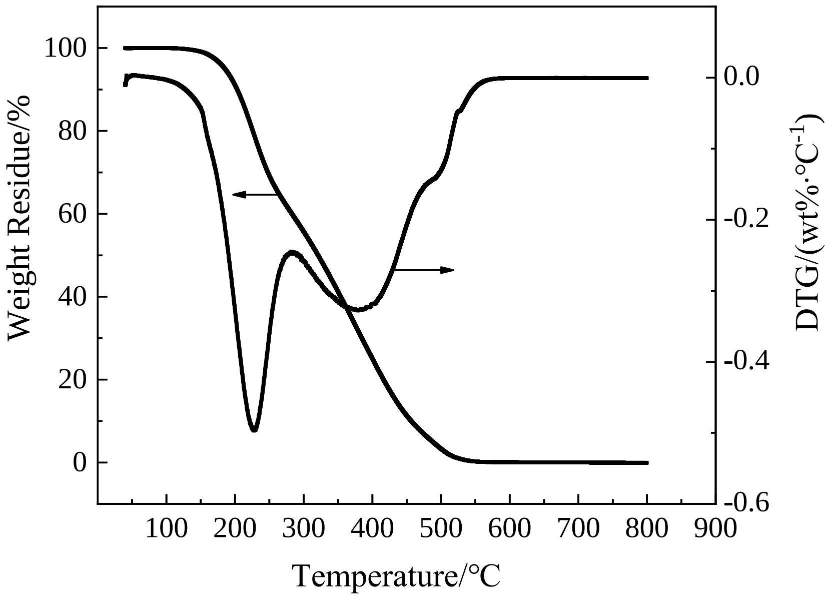

Figure S16. TGA and DTG curves of TMSM-PTFPMS-DMS.

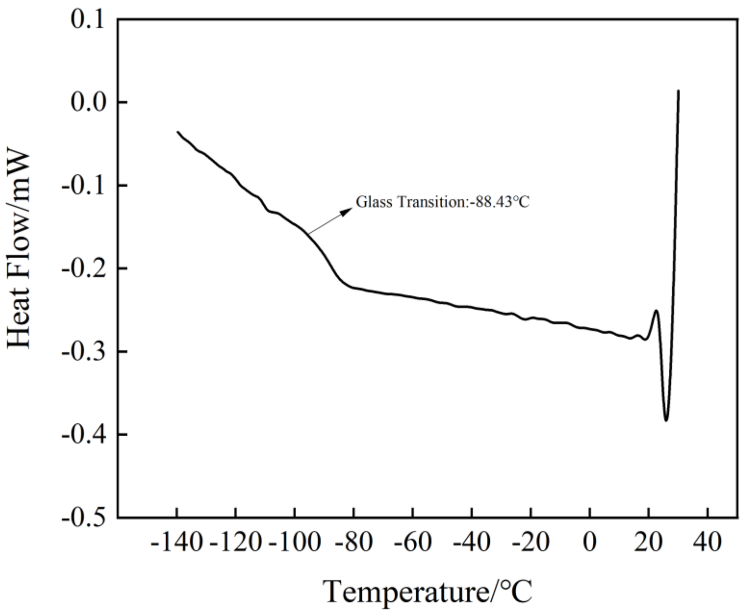

Figure S17. DSC curve of TMSM-PTFPMS-DMS.

The temperatures at $T_{5\%}$, $T_{10\%}$ and T_{max} of TMSM-PTFPMS-DMS in Figure S16 in the Supporting Information were 185.4, 203.3 and 240.2 °C, respectively. As the temperature increased, the TMSM-PTFPMS-DMS sample exhibited a small amount of thermal degradation behavior at around 120 °C. When the temperature rose to about 200 °C, the polymer showed the largest thermal degradation with the maximum thermal degradation rate of −0.52 wt%/°C. When the temperature continued to rise to about 400 °C and 500 °C, a thermal degradation peak and plateau peak appeared in the thermal degradation curve of the polymer, and the corresponding thermal degradation rates were −–0.35 wt%/°C and –0.16 wt%/°C, respectively.

The sample was subjected to a DSC test. During the test, the temperature was raised from −140 °C to 25 °C at a rate of 10 °C/min, and the DSC curve was obtained as shown in Figure S17 in the Supporting Information. It could be seen from the DSC curve that the sample had good structural stability in the range of about −80 °C to 20 °C, and there were no crystallization peaks and melting peaks in this range. When the temperature was reduced to −88.43 °C, the glass transition temperature of the sample was observed, which was in line with the low-temperature resistance characteristic of fluorine functional groups in the polymer molecule.

Like TMSM-PDMS-DMS, the sample was tested using GPC with the same PDMS sample as the reference substance and toluene as the mobile phase. The measured weight average molar mass (*M*w) of the sample was 1051 g/mol, the number average molar mass (*M*n) was 857 g/mol and the polydispersity index (PDI) was 1.23. It can also be observed from the GPC curve (Figure S18 in Supporting Information) that there was only one peak in the GPC curve, which obviously exhibited the characteristics of a normal distribution of high molecular weight polymer and proves the sample was a single component polymer. TLC was used to further confirm that the product was substantially a single component. Because the PDI value was small (1.23) and the peak in the GPC curve was narrow and sharp, it can be concluded that the prepared polymer had a concentrated molecular weight distribution and possessed the characteristics of a narrow molecular weight distribution polymer.

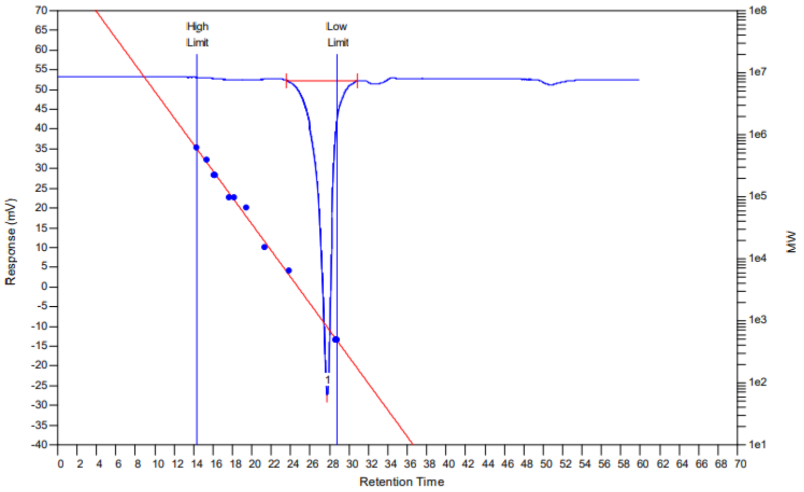

Figure S18. GPC curve of TMSM-PTFPMS-DMS.

2.2. TMSM-PTFPMS-TMOS

The ^{1}H NMR, ^{13}C NMR, ^{29}Si NMR, ^{19}F NMR and FT-IR spectra of TMSM-PTFPMS-TMOS were shown in Figures S19 to S23 in the Supporting information and their characterization information was listed below.

^{1}H NMR (400 MHz, Chloroform-*d*): δ = 3.80–3.22 ppm (m, 8H), 2.27–1.76 ppm (m, 27H), 0.96–0.63 ppm (m, 26H), 0.64–0.45 ppm (m, 3H), 0.34–−0.05 ppm (m, 47H), −0.13 ppm (s, 2H). ^{13}C NMR (126 MHz, Chloroform-*d*): δ = 132.48–123.16 ppm (m), 52.91–46.28 ppm (m), 30.54–25.77 ppm (m), 12.27–6.61 ppm (m), 4.81 ppm (d, J = 67.0 Hz), 2.21–−0.25 ppm (m), 0.19 –−2.79 ppm (m). ^{29}Si NMR (99 MHz, Chloroform-*d*): δ = 15.50–5.05 ppm (m), –0.71 ppm (d, J = 2.8 Hz), −16.85–−27.29 ppm (m), −37.61–−45.42 ppm (m). ^{19}F NMR (471 MHz, Chloroform-*d*): δ = −68.59–−69.01 ppm (m). FT-IR: 1130–1100 cm^{-1} (Si–O–Si), 2964 cm^{-1} (C–H), 1261–787 cm^{-1} (Si–CH_3), 1206 cm^{-1} (CF_3), 1369 cm^{-1} (CH_2CH_2), 2840 cm^{-1} (Si–OCH_3).

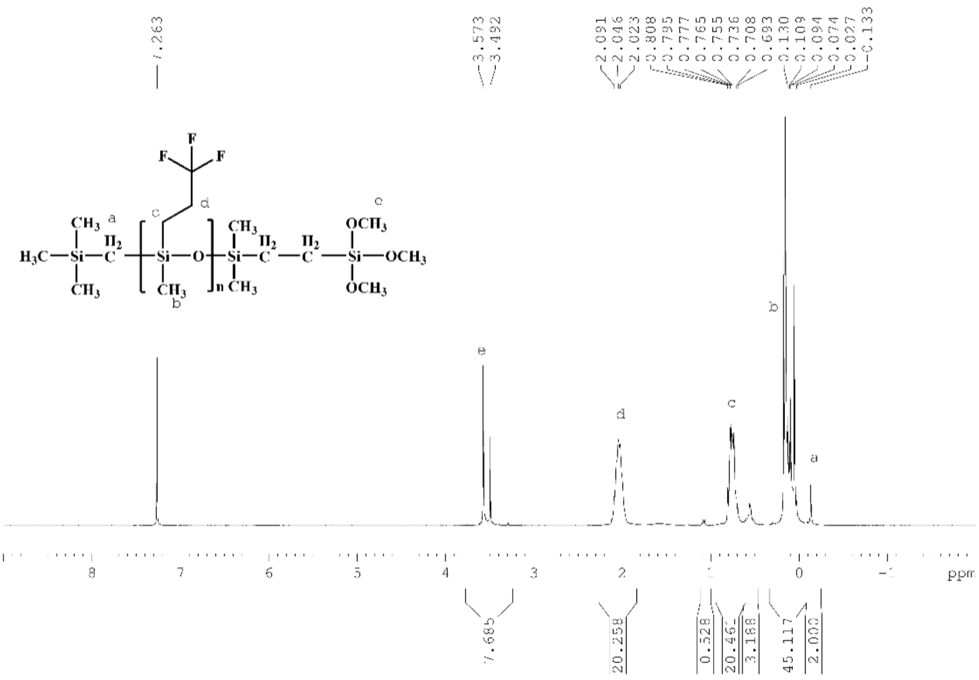

Figure S19. ^{1}H NMR spectra of TMSM-PTFPMS-TMOS.

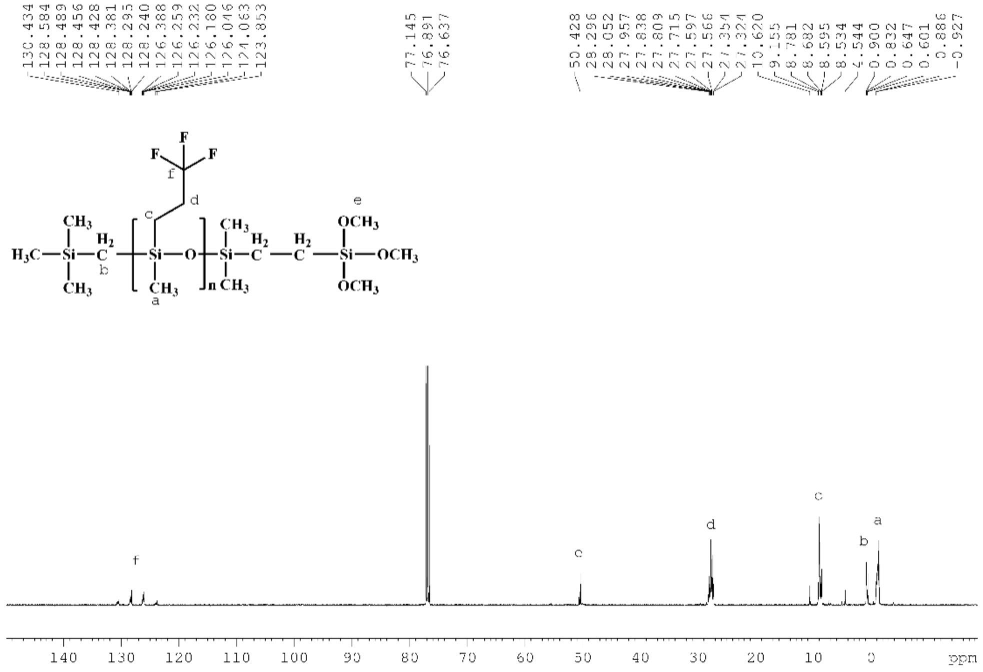

Figure S20. ^{13}C NMR spectra of TMSM-PTFPMS-TMOS.

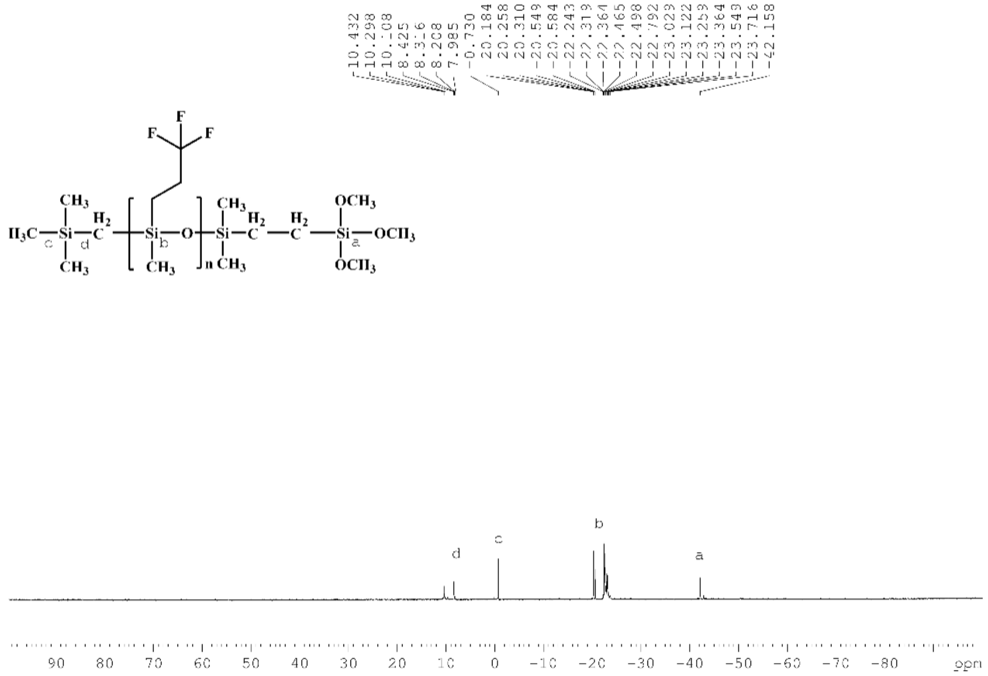

Figure S21. ^{29}Si NMR spectra of TMSM-PTFPMS-TMOS.

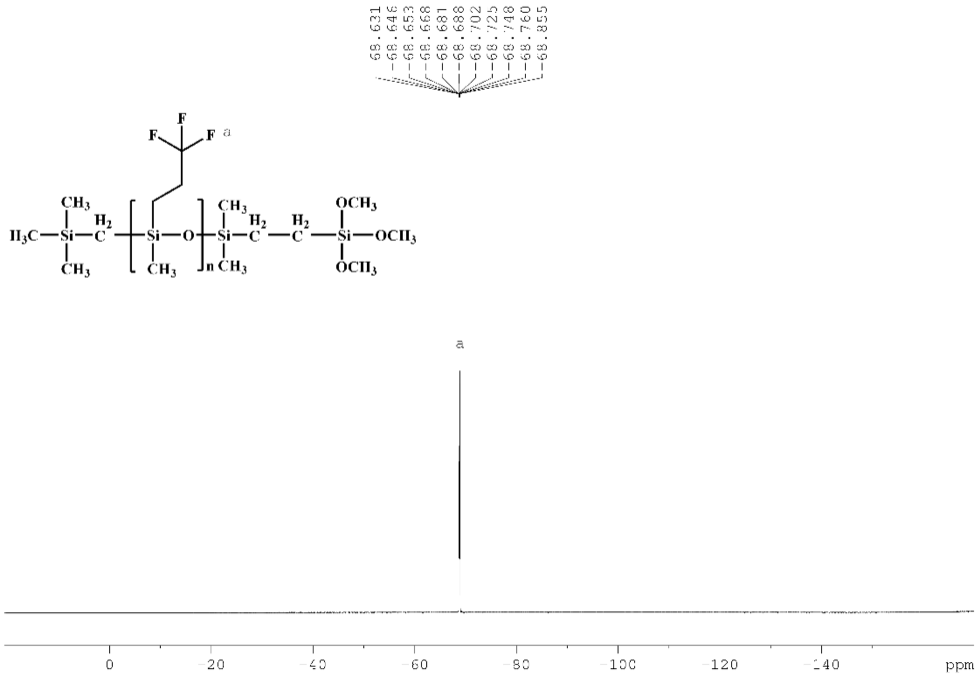

Figure S22. ^{19}F NMR spectra of TMSM-PTFPMS-TMOS.

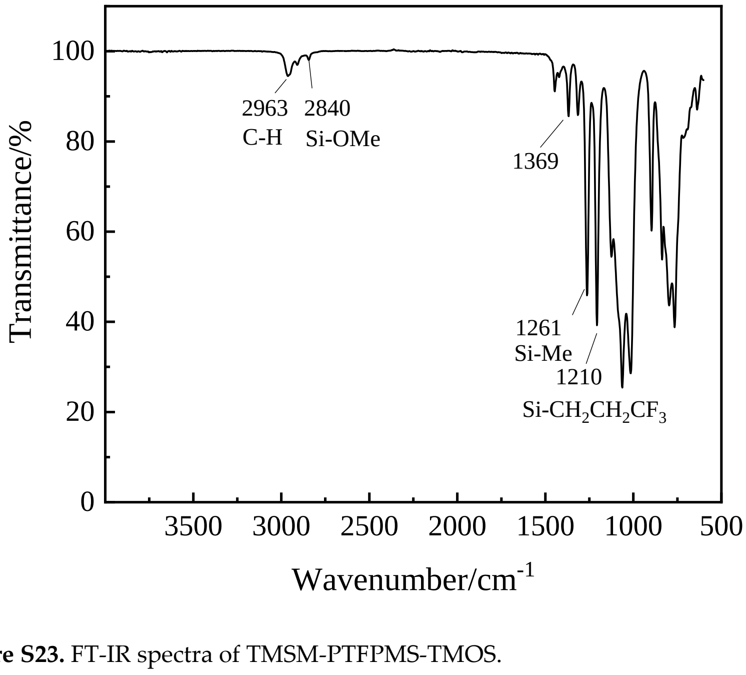

Figure S23. FT-IR spectra of TMSM-PTFPMS-TMOS.

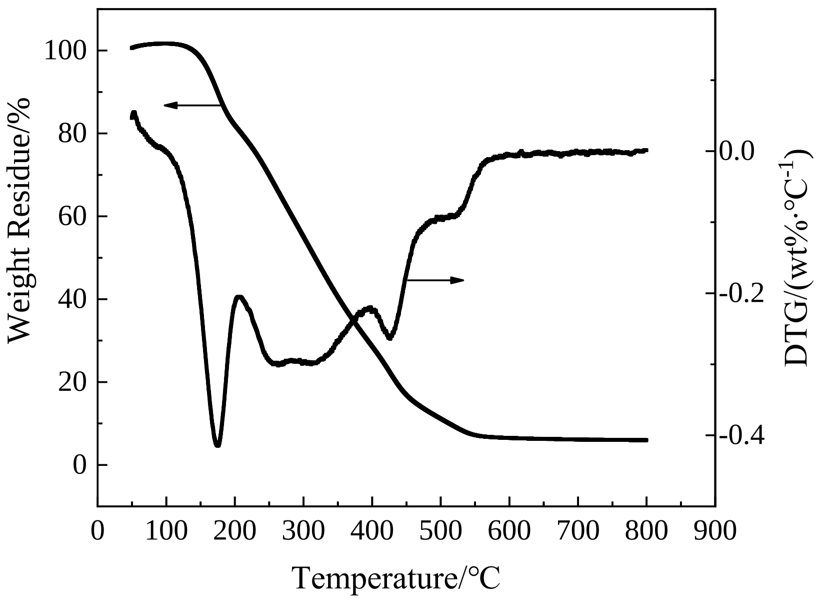

Figure S24. TGA and DTG curves of TMSM-PTFPMS-TMOS.

The temperatures at $T_{5\%}$, $T_{10\%}$ and T_{max} of TMSM-PTFPMS-TMOS in Figure S2**4** in the Supporting Information were 162.0, 174.9 and 180.2 °C, respectively. Because of the presence of fluorine-containing functional groups in the sample, its thermal performance was poor. As the temperature increases, the sample showed a small amount of thermal degradation behavior at about 100 °C. When the temperature rose to about 180 °C, the polymer showed the largest thermal degradation with the maximum thermal degradation rate of −0.45 wt%/°C. When the temperature rose to about 250 °C, there was a thermal degradation peak in the thermal degradation curve of the polymer, which might be the breakage of the Si–$CH_2CH_2CF_3$ bond. When the temperature continued to rise to about 450 °C and 500 °C, a thermal degradation peak and a plateau peak appeared in the thermal degradation curve of the polymer, and the corresponding thermal degradation rates were −0.30 wt%/°C and −0.11 wt%/°C, respectively.

3. TMSM-PDES-TMOS

3.1. TMSM-PDES-DMS

The ^{1}H NMR, ^{13}C NMR, ^{29}Si NMR, ^{19}F NMR and FT-IR spectra of TMSM-PDES-DMS were shown in Figures S2**5** to S2**8** in the Supporting information and their characterization information was listed below.

^{1}H NMR (400 MHz, Chloroform-*d*): δ = 4.73 ppm (s, *J* = 3.0 Hz, 1H), 0.94 ppm (tt, *J* = 8.1, 2.4 Hz, 35H), 0.70–0.30 ppm (m, 24H), 0.19 (d, *J* = 2.8 Hz, 7H), 0.03 ppm (d, *J* = 4.8 Hz, 12H), −0.18 ppm (d, *J* = 1.4 Hz, 2H). ^{13}C NMR (126 MHz, Chloroform-*d*): δ = 54.81 ppm, 9.05 ppm (d, *J* = 1.5 Hz), 8.21–5.35 ppm (m), 3.30–−0.92 ppm (m), –3.57 ppm (d, *J* = 2.3 Hz). ^{29}Si NMR (99 MHz, Chloroform-*d*): δ = 8.56 ppm (d, *J* = 25.8 Hz), –0.55 ppm (d, *J* = 48.9 Hz), −5.77–−13.45 ppm (m), −17.22–−30.69 ppm (m). FT-IR (KBr): 1130–1100 cm^{-1} (Si–O–Si), 2954 cm^{-1} (C–H), 1261–787 cm^{-1} (Si–CH_3), 1240 cm^{-1} (Si–Et), 2127 cm^{-1} (Si–H).

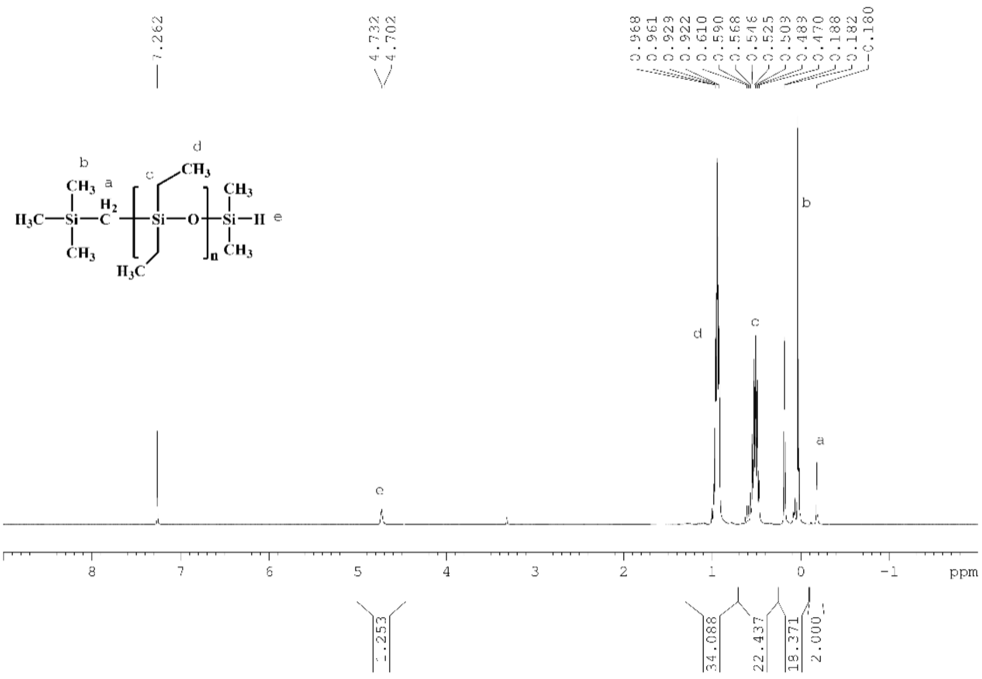

Figure S25. ^{1}H NMR spectra of TMSM-PDES-DMS.

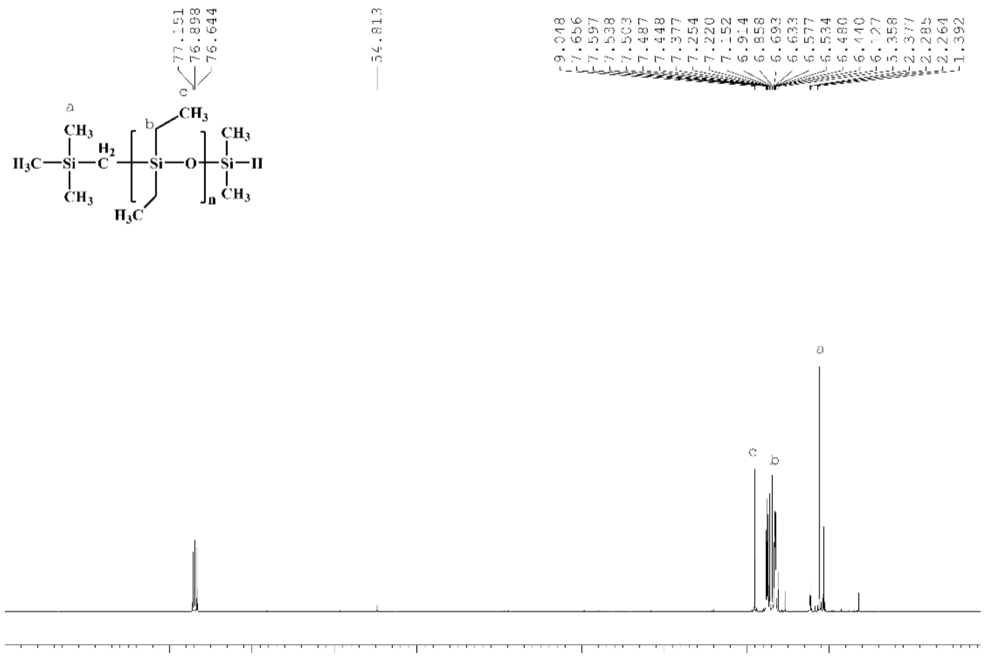

Figure S26. ^{13}C NMR spectra of TMSM-PDES-DMS.

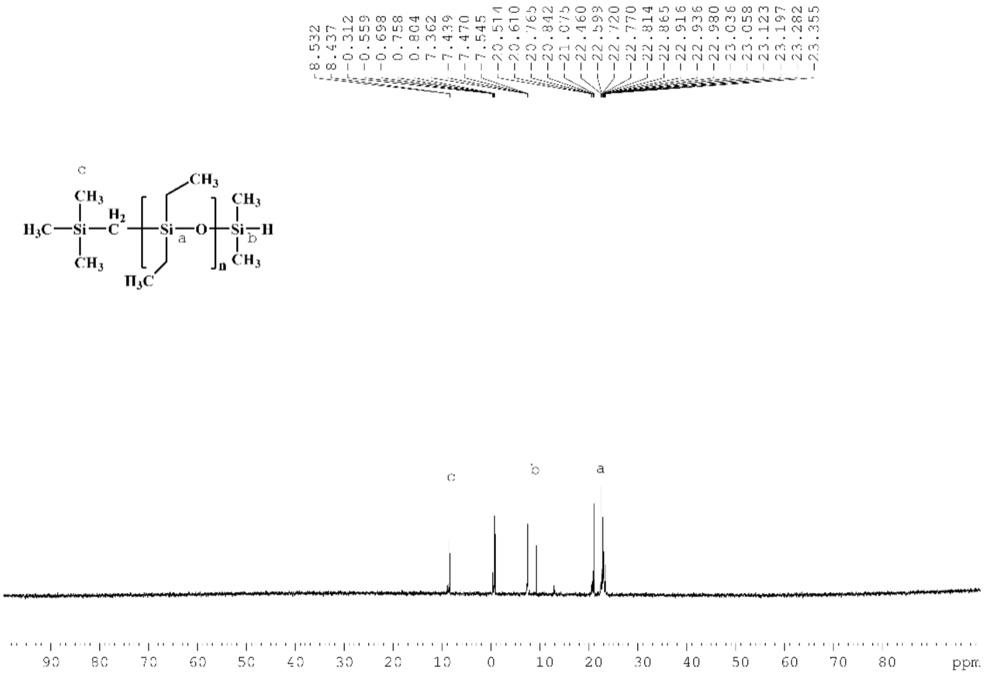

Figure S27. ^{29}Si NMR spectra of TMSM-PDES-DMS.

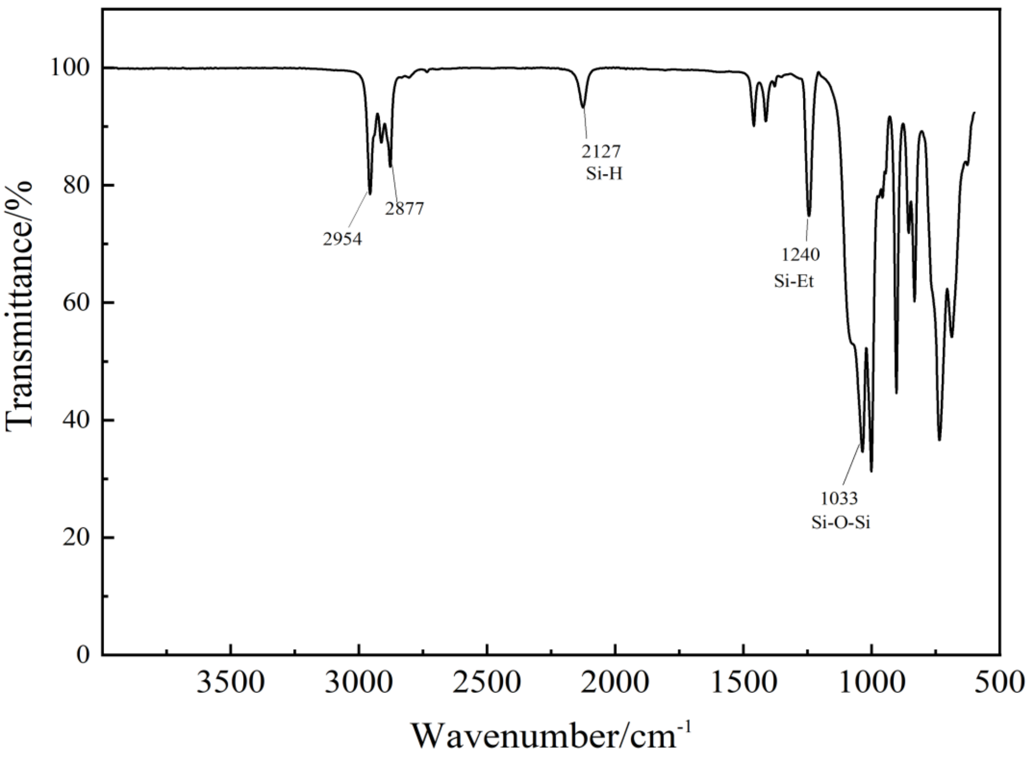

Figure S28. FT-IR spectra of TMSM-PDES-DMS.

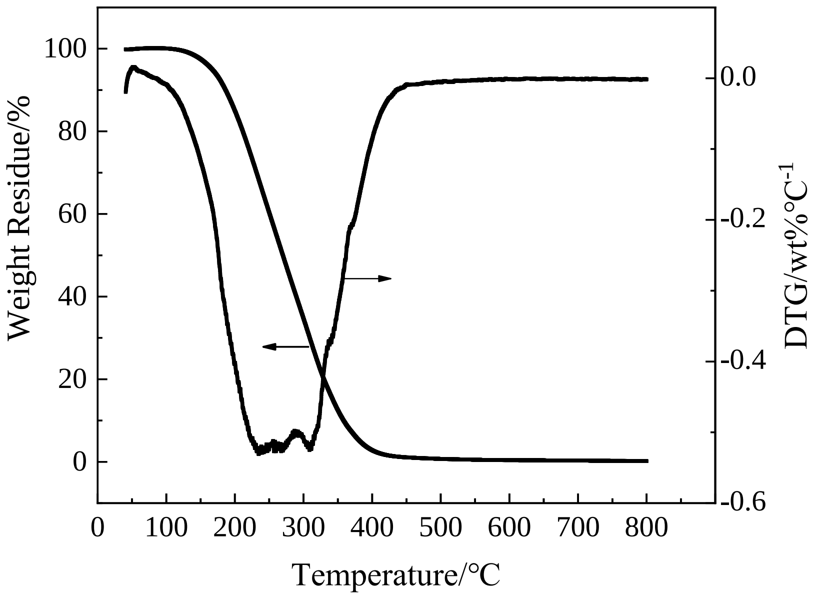

Figure S29. TGA and DTG curves of TMSM-PDES-DMS.

The temperatures at $T_{5\%}$, $T_{10\%}$ and T_{max} of TMSM-PDES-DMS in Figure S2**9** in the Supporting Information were 166.9, 186.5 and 300.3 °C, respectively. As the temperature increased, the sample showed a small amount of thermal degradation behavior at about 100 °C. When the temperature rose to about 300 °C, the largest thermal degradation process was observed in the polymer with a maximum thermal degradation rate of −0.52 wt%/°C.

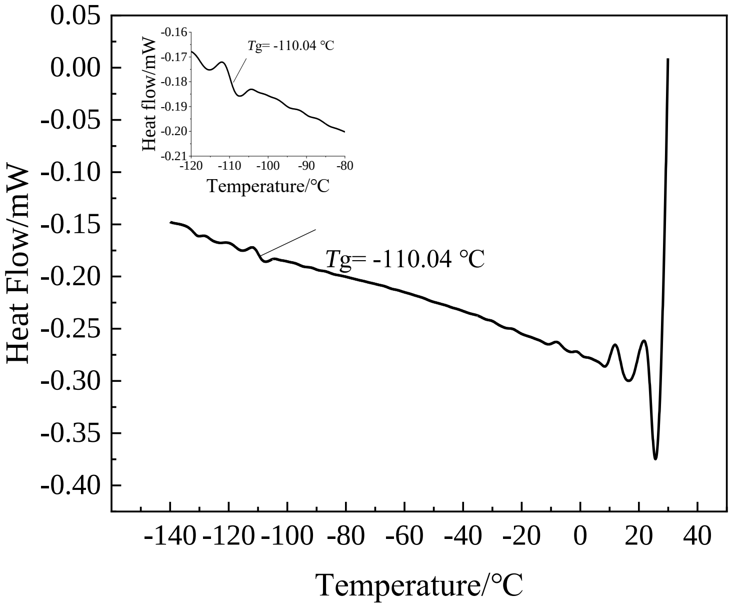

Figure S30. DSC curve of TMSM-PDES-DMS.

The sample was subjected to a DSC test and its DSC curve was shown in Figure S**30** in the Supporting Information. It could be seen from the curve that the sample maintained good structural stability in the range of about −110 °C to 20 °C, and no obvious crystallization peaks and melting peaks could be observed in the above temperature range. When the temperature dropped to −110.04 °C, the glass transition temperature of the sample was observed. This was consistent with the low-temperature resistance characteristics presented by the ethyl functional group in the polysiloxane structure.

Like TMSM-PDMS-DMS, the sample was tested using GPC with the same PDMS sample as the reference substance and toluene as the mobile phase. The measured weight average molar mass (*M*w) of the sample was 879 g/mol, the number average molar mass (*M*n) was 718 g/mol and the polydispersity index (PDI) was 1.22. It could also be observed from the GPC curve (Figure S31 in Supporting Information) that there was only one peak in the GPC curve, which obviously exhibited the characteristics of a normal distribution of high molecular weight polymer and proves the sample was a single component polymer. TLC was used to further confirm that the product was substantially a single component. Because the PDI value was small (1.22) and the peak in the GPC curve was narrow and sharp, it could be concluded that the prepared polymer had a concentrated molecular weight distribution and possessed the characteristics of a narrow molecular weight distribution polymer.

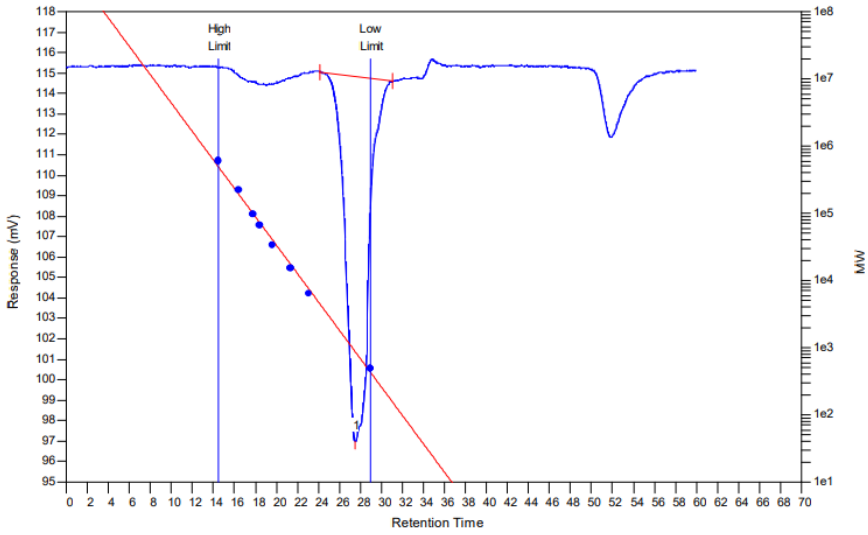

Figure S31. GPC curve of TMSM- PDES -DMS.

3.2. TMSM-PDES-TMOS

The ^{1}H NMR, ^{13}C NMR, ^{29}Si NMR, ^{19}F NMR and FT-IR spectra of TMSM-PDES-TMOS were shown in Figures S32 to S35 in the Supporting information and their characterization information was listed below.

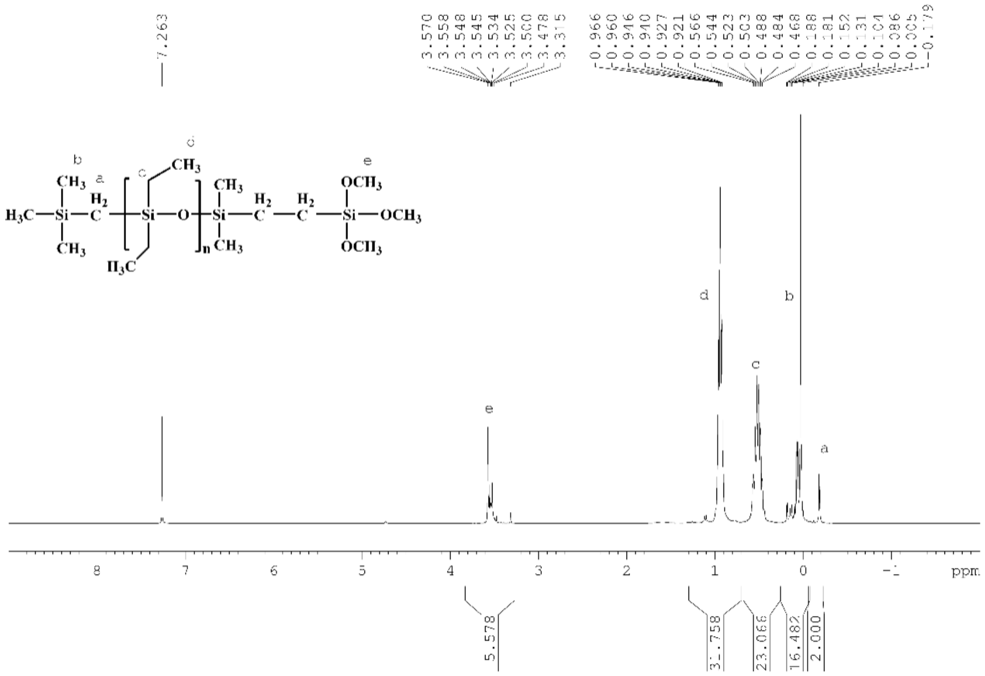

Figure S32. ^{1}H NMR spectra of TMSM-PDES-TMOS.

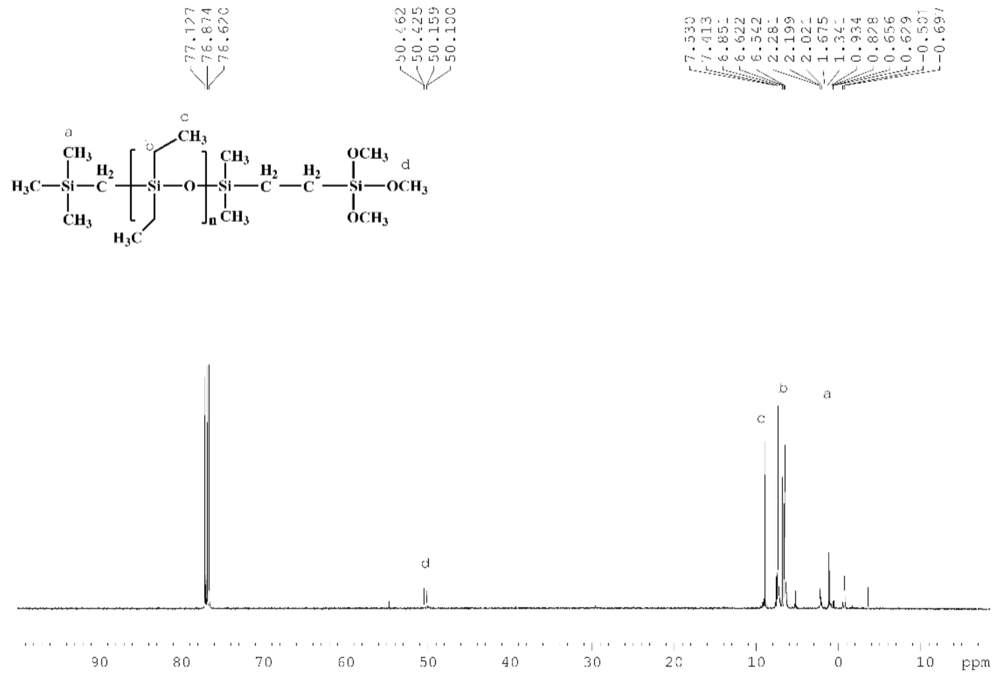

Figure S33. ^{13}C NMR spectra of TMSM-PDES-TMOS.

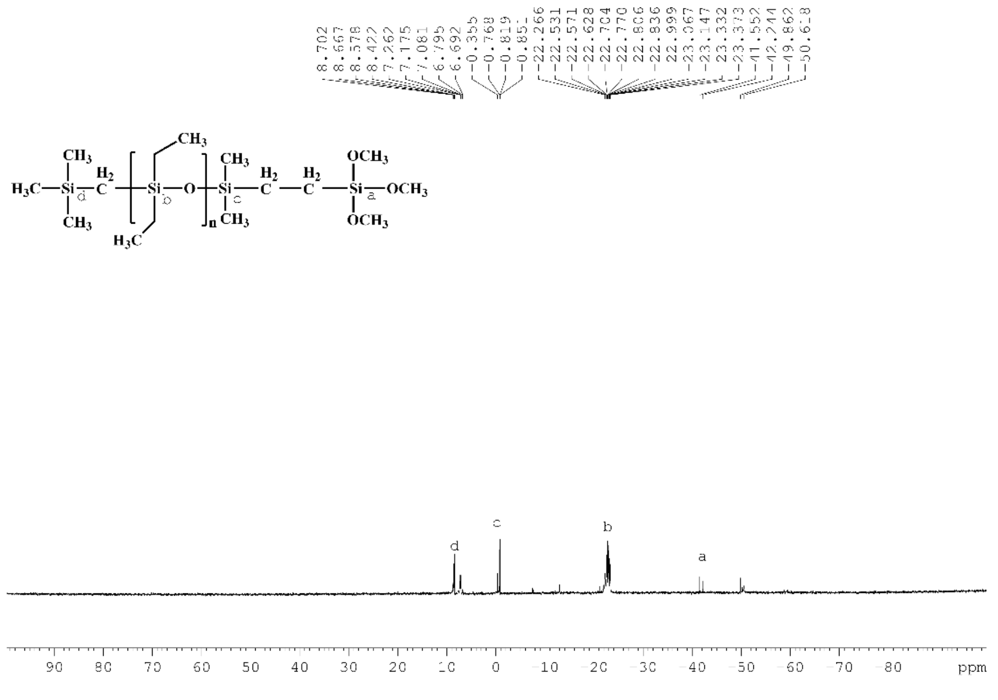

Figure S34. ^{29}Si NMR spectra of TMSM-PDES-TMOS.

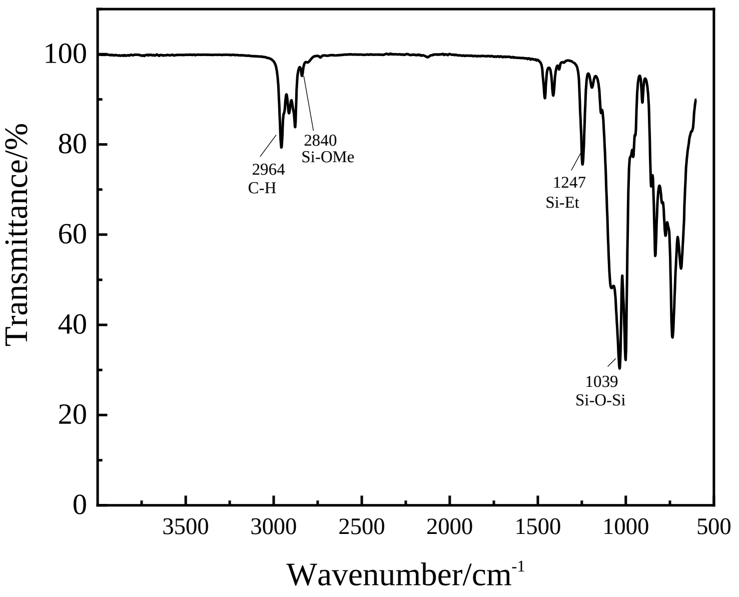

Figure S35. FT-IR spectra of TMSM-PDES-TMOS.

^{1}H NMR (400 MHz, Chloroform-*d*): δ= 3.67–3.45 ppm (m, 6H), 0.94 ppm (tt, *J* = 8.1, 4.2 Hz, 36H), 0.70–0.36 ppm (m, 27H), 0.24–−0.06 ppm (m, 18H), −0.18 ppm (d, *J* = 2.6 Hz, 2H). ^{13}C NMR (101 MHz, Chloroform-*d*): δ= 51.14–49.37 ppm (m), 8.92 ppm, 8.01–5.82 ppm (m), 2.22 ppm (d, *J* = 8.1 Hz), 1.14 ppm, −0.73 ppm, −3.59 ppm. ^{29}Si NMR (99 MHz, Chloroform-*d*): δ= 9.69–7.81 ppm (m), 7.81–6.04 ppm (m), 0.94–−1.95 ppm (m), −20.29–−24.74 ppm (m), −41.89 ppm (d, *J* = 68.9 Hz), −48.98–−50.86 ppm (m). FT-IR (KBr): 1130–1100 cm^{-1} (Si–O–Si), 2954 cm^{-1} (C–H), 1261–787 cm^{-1} (Si–CH_3), 1240 cm^{-1} (Si–Et), 2840 cm^{-1} (Si–OCH_3).

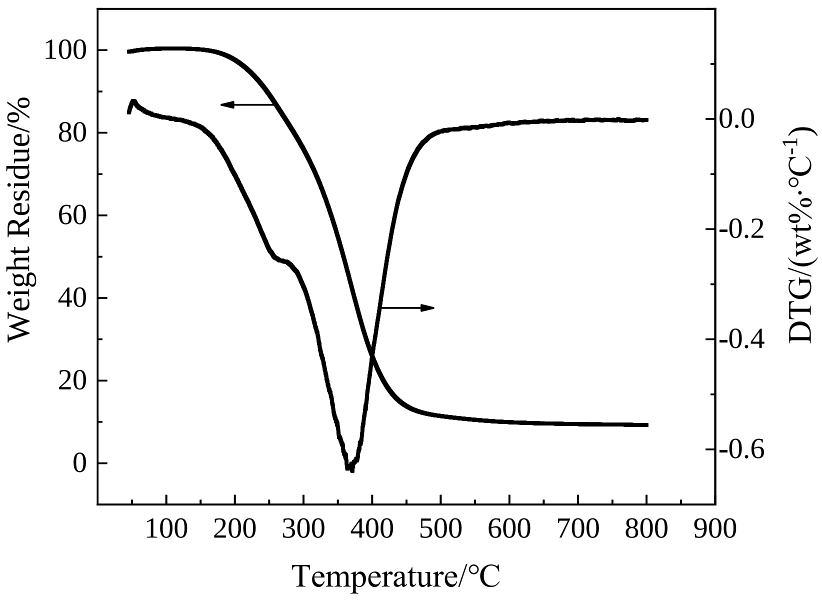

Figure S36. TGA and DTG curves of TMSM-PDES-TMOS.

The temperatures at $T_{5\%}$, $T_{10\%}$ and T_{max} of TMSM-PDES-TMOS in Figure S3**6** in the Supporting Information were 220.6, 247.0 and 360.1 °C, respectively. As the temperature increased, the sample showed a small amount of thermal degradation behavior at about 120 °C. When the temperature rose to about 360 °C, the largest thermal degradation occurred with the maximum thermal degradation rate of −0.62 wt%/°C.
